# Apoptotic brown adipocytes enhance energy expenditure via extracellular inosine

**DOI:** 10.1038/s41586-022-05041-0

**Published:** 2022-07-05

**Authors:** Birte Niemann, Saskia Haufs-Brusberg, Laura Puetz, Martin Feickert, Michelle Y. Jaeckstein, Anne Hoffmann, Jelena Zurkovic, Markus Heine, Eva-Maria Trautmann, Christa E. Müller, Anke Tönjes, Christian Schlein, Azin Jafari, Holger K. Eltzschig, Thorsten Gnad, Matthias Blüher, Natalie Krahmer, Peter Kovacs, Joerg Heeren, Alexander Pfeifer

**Affiliations:** 1 Institute of Pharmacology and Toxicology, University Hospital, University of Bonn, Bonn, Germany; 2 Department of Biochemistry and Molecular Cell Biology, University Medical Center Hamburg-Eppendorf, Hamburg, Germany; 3 Helmholtz Institute for Metabolic Obesity and Vascular Research (HI-MAG) of the Helmholtz Zentrum München at the University of Leipzig and University Hospital Leipzig, Leipzig, Germany; 4 Institute for Diabetes and Obesity, Helmholtz Center Munich, Neuherberg, Germany; 5 Pharmaceutical Institute, Pharmaceutical & Medicinal Chemistry, University of Bonn, Bonn, Germany; 6 PharmaCenter Bonn, University of Bonn, Bonn, Germany; 7 Medical Department III – Endocrinology, Nephrology, Rheumatology, University of Leipzig Medical Center, Leipzig, Germany; 8 Institute of Human Genetics, University Medical Center Hamburg-Eppendorf, Hamburg, Germany; 9 Clinic and Polyclinic for General, Visceral, Thoracic and Vascular Surgery, University Hospital, University of Bonn, Bonn, Germany; 10 Department of Anesthesiology, University of Texas Health Science Center at Houston, McGovern Medical School, Houston, Texas USA; 11 German Center for Diabetes Research (DZD), Neuherberg, Germany

**Keywords:** Fat metabolism, Extracellular signalling molecules

## Abstract

Brown adipose tissue (BAT) dissipates energy^1,2^ and promotes cardiometabolic health^3^. Loss of BAT during obesity and ageing is a principal hurdle for BAT-centred obesity therapies, but not much is known about BAT apoptosis. Here, untargeted metabolomics demonstrated that apoptotic brown adipocytes release a specific pattern of metabolites with purine metabolites being highly enriched. This apoptotic secretome enhances expression of the thermogenic programme in healthy adipocytes. This effect is mediated by the purine inosine that stimulates energy expenditure in brown adipocytes by the cyclic adenosine monophosphate–protein kinase A signalling pathway. Treatment of mice with inosine increased BAT-dependent energy expenditure and induced ‘browning’ of white adipose tissue. Mechanistically, the equilibrative nucleoside transporter 1 (ENT1, SLC29A1) regulates inosine levels in BAT: ENT1-deficiency increases extracellular inosine levels and consequently enhances thermogenic adipocyte differentiation. In mice, pharmacological inhibition of ENT1 as well as global and adipose-specific ablation enhanced BAT activity and counteracted diet-induced obesity, respectively. In human brown adipocytes, knockdown or blockade of ENT1 increased extracellular inosine, which enhanced thermogenic capacity. Conversely, high *ENT1* levels correlated with lower expression of the thermogenic marker *UCP1* in human adipose tissues. Finally, the Ile216Thr loss of function mutation in human *ENT1* was associated with significantly lower body mass index and 59% lower odds of obesity for individuals carrying the Thr variant. Our data identify inosine as a metabolite released during apoptosis with a ‘replace me’ signalling function that regulates thermogenic fat and counteracts obesity.

## Abstract (web summary)

Untargeted metabolomics demonstrate that apoptotic brown adipocytes release a specific pattern of metabolites with purine metabolites being highly enriched, and inosine is identified as a metabolite released during apoptosis regulating thermogenic fat and counteracting obesity.

## Main

In contrast to white fat that stores energy, brown adipose tissue (BAT) dissipates energy and has been shown to promote cardiometabolic health in humans^3^. Adipose tissues adapt to the nutritional/metabolic state and show an intriguing plasticity that requires precise regulation of proliferation as well as of apoptosis^4^. In BAT, apoptosis is a continuing process and chronic inactivation (for example, thermoneutrality or denervation) results in reduced activity and abundance of brown adipocytes^5–8^. Obesity and ageing are associated with a functional atrophy of BAT and impairment of adaptive thermogenesis in humans^9,10^. However, the signals released by apoptotic brown adipocytes have so far not been investigated. Nucleosides are biologically important molecules that serve many purposes including synthesis of nucleic acids and energy metabolism (adenosinetriphosphate (ATP)). Moreover, purinergic molecules also have functions as signalling molecules and ATP is released by dying cells as danger signal^11^.

## Secretome of apoptotic brown adipocytes

**Fig. 1:**
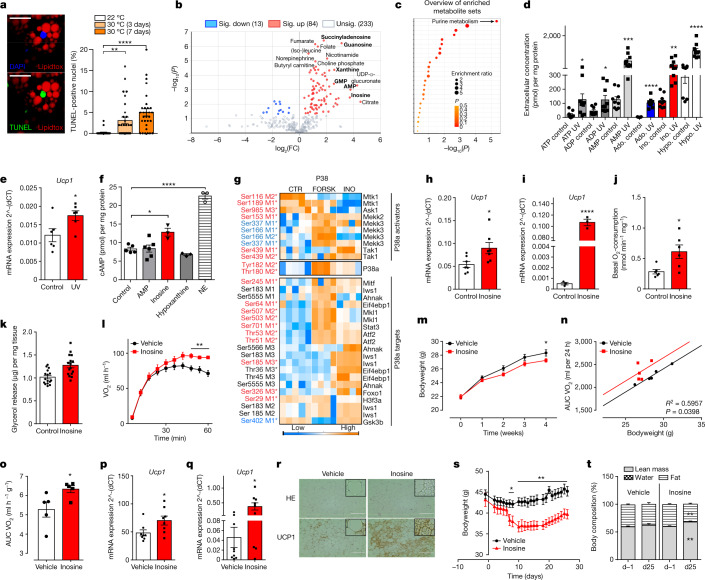
Stimulatory effects of inosine on BAT metabolism. **a**, Left, representative image of BAT after thermoneutrality: whole mount staining of lipids (LipidTOX, red), apoptotic nuclei (TUNEL-stain, green) and nuclei (DAPI, blue). Right, quantification of TUNEL-positive nuclei in BAT at 22 or 30 °C for 3 or 7 days (*n* = 3, 10 images per BAT). Scale bar, 20 µm. **b**,**c**, Untargeted metabolomics of murine brown adipocytes after nutlin-3 treatment (*n* = 6): **b**, Volcano plot representing statistically increased (red) or reduced (blue) metabolites. Sig., significant. **c**, Qualitative enrichment analysis of metabolic pathways (also see Supplementary Fig. 1). **d**, Extracellular purinergic molecules after UV irradiation of brown adipocytes (*n* = 9). ADP, adenosine diphosphate; Ado, adenosine; AMP, adenosine monophosphate; Hypo, hypoxanthine; Ino, inosine. **e**, Expression of *Ucp1* in brown adipocytes after incubation with supernatants described in **d** (*n* = 6). **f**, Intracellular cAMP levels of murine brown adipocytes treated with indicated compounds (*n* = 3–6). **g**, Hierarchical clustering of P38 signalling regulated by inosine (INO) and FORSK. Regulatory sites: *Activating sites, red; inhibitory sites, blue. **h**,**i**, Expression of *Ucp1* in murine brown adipocytes (**h**) (*n* = 7) and white adipose (**i**) (*n* = 3) after inosine treatment. **j**,**k**, Basal oxygen consumption (*n* = 6) (**j**) and lipolysis (*n* = 16) (**k**) of BAT explants after inosine treatment. **l**, Oxygen consumption of mice after inosine injection (*n* = 5). **m**–**r**, Inosine administration via micro-osmotic pumps and HFD for 28 days. **m**, Body weight (*n* = 9–10). **n**, Analysis of covariance (ANCOVA) (non-linear fit) area under the curve (AUC) of oxygen consumption/body weight at 23 °C (*n* = 5). **o**, Oxygen consumption at 4 °C (*n* = 5). **p**,**q**, *Ucp1* expression in BAT (*n* = 8) (**p**) and WATi (*n* = 9) (**q**). **r**, Representative haematoxylin and eosin (HE) and UCP1 staining of WATi (HE *n* = 8, UCP1 *n* = 3, Scale bar, 100 µm; upper right, fourfold magnification). **s**,**t**, Inosine injections in obese mice (vehicle: *n* = 8, inosine: *n* = 7). **s**, Body weight. **t**, Body composition at days −1 and 25. For all: *P < 0.05, **P < 0.01, ***P < 0.005, ****P < 0.001. For exact *P* values, see source data. Data are represented as mean ± s.e.m. Two-tailed *t*-test, **d**,**e**,**h–m**,**o**–**q**; one-way ANOVA with Tukey’s post hoc test, **a**–**c**,**f**. Source data

**Extended Data Fig. 1:**
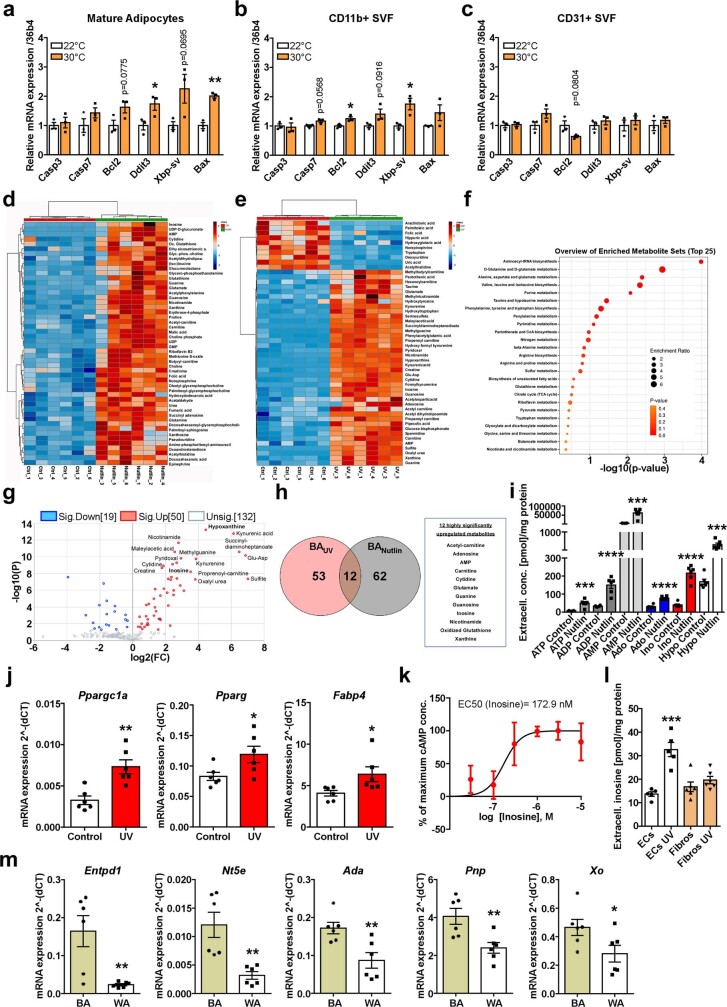
Secreted factors of apoptotic murine brown adipocytes. **a–c**, Mature adipocytes, CD11b+ myeloid cells and CD31+ endothelial cells were isolated from BAT of mice housed at 22 °C or at 30 °C (thermoneutrality) for 3 days. Gene expression of apoptotic marker genes in (**a**) mature adipocytes, (**b**) CD11b+ myeloid cells, (**c**) CD31+ endothelial cells (n = 3). **d–h**, Untargeted metabolomics of apoptotic BA. **d**, Heatmap of top 50 secreted metabolites accumulated in the supernatant after nutlin-3 treatment, upregulated metabolites: red, downregulated metabolites: blue (n = 6). **e**, Heatmap of top 50 secreted metabolites after UV irradiation (n = 6). **f**, Qualitative enrichment analysis of metabolic pathways of secreted metabolites of BA after UV irradiation based on the KEGG metabolic pathways, **g**, Volcano plot of secreted metabolites after UV treatment of BA (n = 6). **h**, Venn diagram and shared metabolite list identified across all highly significantly (p < 0.01) up- or down-regulated metabolites after UV or nutlin-3 treatment. **i**, Extracellular concentrations of purinergic molecules of BA after nutlin-3 treatment (n = 6). **j**, Expression of *Ppargc1a, Pparg* and *Fabp4* in BA after treatment with the apoptotic supernatant described in Fig. 1d (n = 6). **k**, cAMP dose-response curve of inosine (n = 3). **l**, Extracellular inosine concentrations of murine mesenchymal endothelial cells (EC) and murine BAT-derived fibroblasts (Fibros) after UV irradiation (n = 5-6). **m**, Expression of the ATP-degrading enzymes *Entpd1, Nt5e, Ada, Pnp, Xo* in murine brown (BA) and white (WA) adipocytes (n = 6). For all: *p < 0.05, **p < 0.01, ***p < 0.005, ****p < 0.001. For exact p-values see Source Data. Data are represented as mean ± s.e.m. Two-tailed t-test was applied for **a–c**, **i**, **j**, **l**, **m**; One-way ANOVA with Tukey’s post hoc test for **g**, **h**. Source data

To study whether apoptosis naturally occurs to an appreciable degree under physiological conditions, we housed mice at thermoneutrality (30 °C) to inactivate BAT. After 3 and 7 days of thermoneutral housing, we observed a significant increase in apoptotic, TUNEL-positive cells in BAT (Fig. 1a). To define the cell types undergoing apoptosis, we isolated mature adipocytes, CD11b-positive immune cells and CD31-positive endothelial cells from BAT of mice housed at 30 °C. Compared to controls (22 °C), we found increased expression of apoptotic markers^12^ predominantly in mature adipocytes (Extended Data Fig. 1a–c), including the pro-apoptotic transcription factors DNA damage-inducible transcript 3 (Ddit3), the spliced messenger RNA form of the X-box binding protein 1 (Xbp1-sv) and the BCL2 associated X protein (Bax). Notably, an independent study^13^ using an elegant cell-type-specific approach demonstrated a similar apoptotic signature in brown adipocytes even after exposure to thermoneutrality for 4 weeks. These findings suggest that physiological inactivation of BAT induces an adaptive physiological response that promotes apoptosis particularly in thermogenic adipocytes.

Next, we used an untargeted comparative metabolomic approach to identify metabolites involved in apoptosis of brown adipocytes, followed by a targeted approach focusing on the most upregulated metabolic pathway. Murine preadipocytes/stromal vascular fractions were differentiated to mature brown adipocytes and irradiated with ultraviolet (UV) light to induce caspase-dependent apoptosis^14^ or treated with nutlin-3, a small-molecule inhibitor of MDM2, to trigger apoptosis via the p53 pathway^15^. The conditions were optimized so that they significantly induced apoptosis in adipocytes (annexinV-positive cells), while ensuring the integrity of the cell membrane (propidium iodide-negative).

The metabolites in the supernatant of cells were detected against a spectral library of more than 3,500 endogenous metabolites on the basis of, among others, their accurate mass to charge ratio and their fragmentation patterns. Nutlin-3-induced apoptosis resulted in significant enrichment of 84 metabolites, while 13 metabolites were significantly reduced (Fig. 1b). In total, a broad range of 330 compounds were detected in the supernatant of brown adipocytes, with purinergic nucleotides (for example, succinyladenosine, guanosine, inosine) being strongly represented among the most significantly upregulated compounds (Extended Data Fig. 1d). Accordingly, qualitative enrichment analysis of metabolic pathways showed that purine metabolism is the most significantly altered metabolic pathway in brown adipocytes during nutlin-3-induced apoptosis (Fig. 1c and Supplementary Fig. 1).

Similarly, induction of apoptosis by UV light strongly affected the secretome of brown adipocytes (Extended Data Fig. 1e), with significant involvement of the purine metabolic pathway (Extended Data Fig. 1f) and significant up- and down-regulation of 50 and 19 metabolites, respectively (Extended Data Fig. 1g). In both treatments (nutlin-3 or UV), 12 metabolites were highly significantly upregulated, with six of them belonging to the family of purinergic molecules (Extended Data Fig. 1h).

Next, a targeted approach analysing extracellular purinergic molecules was performed focusing on ATP-derived molecules, because ATP has previously been shown to be a major apoptosis-related metabolite in other cell types^11^. During UV-induced apoptosis, brown adipocytes released a specific pattern of purine molecules (Fig. 1d) with significantly increased ATP levels (Fig.1d). However, three other significantly increased purine molecules, AMP, inosine and hypoxanthine, reached the highest extracellular concentrations (Fig. 1d). A similar pattern was observed after nutlin-3 treatment (Extended Data Fig. 1i).

Incubation of healthy brown adipocytes with the supernatant of apoptotic brown fat cells resulted in significantly increased expression of the thermogenic marker genes *Ucp1* and *Ppargc1a* as well as of adipogenic differentiation markers *Pparg* and *Fabp4* (Fig. 1e and Extended Data Fig. 1j) indicating that dying brown adipocytes signal for replacement to maintain tissue function.

## Inosine enhances the thermogenic programme

Given the stimulatory effect of the supernatant from apoptotic brown adipocytes, the concentration of cyclic adenosine monophosphate (cAMP), the central second messenger that enhances differentiation and the thermogenic programme in brown adipocytes^16^, was analysed. To identify individual metabolites that mediate this effect, we focused on the three most abundantly secreted extracellular purines (Fig. 1d). Neither AMP nor hypoxanthine significantly altered cAMP levels (Fig. 1f). By contrast, the purine inosine induced a significant increase in intracellular cAMP (Fig. 1f and Extended Data Fig. 1k) indicating that inosine might play a so far unknown role in BAT activation and metabolism.

To define the source of inosine in BAT, we also measured inosine release in endothelial cells and fibroblasts, which plays an important role in adipose tissue. Endothelial cells showed significantly increased extracellular inosine concentrations after UV irradiation, while the extracellular inosine levels of fibroblasts were not significantly changed (Extended Data Fig. 1l). Both cell types showed lower extracellular inosine concentrations under both basal conditions and after UV irradiation compared to brown adipocytes (Extended Data Fig. 1l and Fig. 1d). These findings indicate that, among the cell types investigated, brown adipocytes are the main source of extracellular inosine in BAT.

Next, we analysed the expression of purinergic enzymes. All main enzymes involved in the ATP degradation cascade including ectonucleoside triphosphate diphosphohydrolase 1 (ENTPD1) and ecto-5′-nucleotidase (NT5E) as well as the enzyme that catalyses the conversion of adenosine to inosine; that is, adenosine deaminase were significantly higher expressed in murine brown adipocytes compared to white adipocytes (Extended Data Fig. 1m).

## Inosine signals by the cAMP–protein kinase A axis

**Extended Data Fig. 2:**
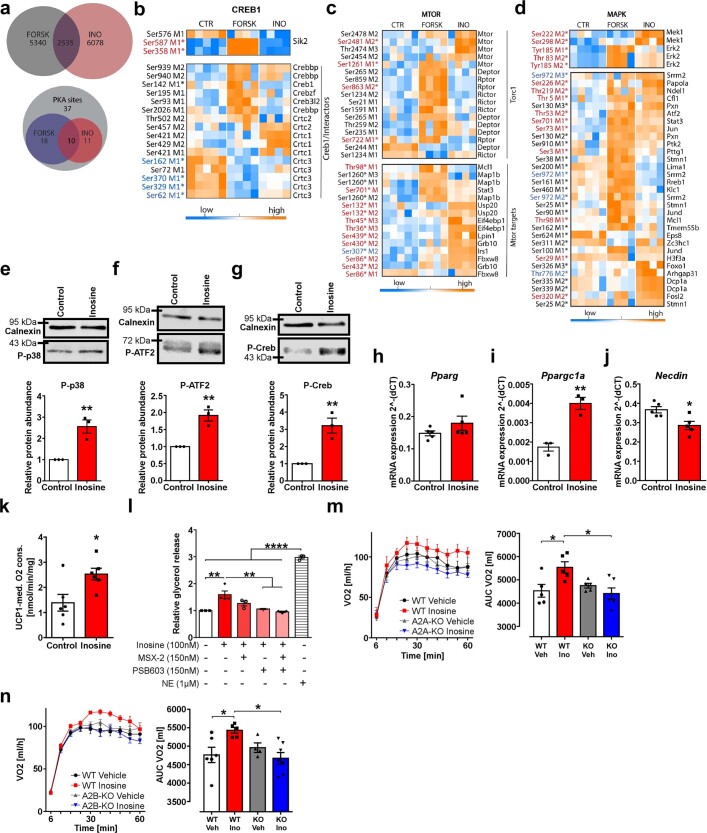
Inosine stimulates thermogenesis. **a**, Venn-Diagram of significantly changed phosphorylation sites upon FORSK or INO treatments (FDR < 0.05) and Venn-diagram indicating significantly regulated sites upon FORSK and INO treatments and total number of PKA target sites with more than 3 valid values in at least one condition. **b–d**, Hierarchical clustering of significantly regulated sites of (**b**) the Sik2-Creb1 axis, (**c**) the Mtorc1 complex and regulatory MTOR target sites and (**d**) regulatory sites of MAPK/ERKs and MAPK target sites, significantly regulated by INO and FORSK. Regulatory sites: *. Activating sites: red, inhibitory sites: blue. **e–g**, Representative Western Blots and quantification of (**e**) P-p38 MAP kinase, (**f**) P-ATF-2 and (**g**) P-Creb in response to 30 min of inosine (300 nM) in murine BA (n = 3). Calnexin serves as a loading control. For gel source data see Supplementary Information. **h**, Expression of *Pparg* mRNA in murine BA inosine treatment (n = 5). **i**, Expression of *Ppargc1a* in murine WA after inosine treatment (n = 3). **j**, Expression of *Necdin* in premature WA after inosine-treatment (n = 5). **k**, UCP1-mediated oxygen consumption of BAT explants after inosine-treatment (n = 6). **l**, Lipolysis of murine BA after 120 min of incubation with solvent control, inosine (100 nM), norepinephrine (NE, 1 µM) with or without pre-treatment with the A_2A_-antagonist MSX (150nM) or the A_2B_-antagonist PSB603 (150 nM) (n = 3-4). **m**, **n**, Oxygen consumption at 23 °C after i.p injection of either Vehicle (Veh) or inosine (Ino) and respective AUCs of measured V(O2) over 60 min of 8-week-old, male WT and (**m**) A_2A_-KO mice (n = 5-6) and (**n**) A_2B_-KO mice (n = 4-7). For all: *p < 0.05, **p < 0.01, ***p < 0.005, ****p < 0.001. For exact p-values see Source Data. Data are represented as mean ± s.e.m. Two-tailed t-test was applied for **e–k**. One-way ANOVA with Tukey’s post hoc test for **l–n**. Source data

The significant increase in intracellular cAMP on inosine treatment prompted us to investigate whether inosine activates the canonical cAMP–protein kinase A (PKA) pathway. A high-sensitivity phosphoproteomic analysis of murine brown adipocytes treated with either inosine or the adenylate cyclase activator forskolin (FORSK) identified 38,451 phosphopeptides. On FORSK and inosine treatment, 7,875 and 8,613 phospho-sites were regulated (FDR < 0.05), respectively, with 2,535 phospho-sites being significantly regulated in both treatments (Extended Data Fig. 2a). PKA target sites were over-represented among the regulatory sites (Extended Data Fig. 2a). In FORSK, as well as in inosine-treated brown adipocytes, we detected an activation of the p38 mitogen-activated protein kinase (MAPK) axis resulting in transcription inducing Thr53 and Thr51 phosphorylation of activating transcription factor 2 (ATF2) (Fig. 1g). Both inosine and FORSK reduced phosphorylation at the regulatory site Ser576 of Salt-inducible kinase 2 (Sik2), resulting in a decreased downstream phosphorylation of cAMP-regulated transcriptional coactivator 3 (Crtc3) (Ser72, Ser162, Ser329, Ser370) (Extended Data Fig. 2b). Dephosphorylation at these sites has previously been shown to induce Crtc3 nuclear import and to promote the cAMP-responsive element binding protein 1 (Creb1) association and activation^17^. In addition, both FORSK and inosine activated mechanistic target of rapamycin (mTOR) complex 1 (mTORC1) and MAPK/extracellular signal-regulated kinases signalling, which have been described to promote brown adipocyte differentiation and browning (Extended Data Fig. 2c,d)^18,19^. Thus, the phosphoproteomics data show that inosine activates PKA signalling and the PKA downstream targets p38, Sik2, Crtc3 and Creb that control the core brown/beige genetic programme and thermogenic capacity^20,21^.

Western blot analyses confirmed the activation of p38 MAPK and of its downstream target ATF2 as well as the activation of Creb by inosine in murine brown adipocytes (Extended Data Fig. 2e–g).

As a consequence, inosine treatment significantly increased *Ucp1* expression in murine brown adipocytes (Fig. 1h) and *Pparg* expression was enhanced by 21%, albeit not significantly (Extended Data Fig. 2h). Inosine-induced browning of mature white adipocytes as indicated by significantly increased expression of the thermogenic genes *Ucp1* and *Ppargc1a* (Fig. 1i and Extended Data Fig. 2i), whereas expression of the adipogenesis-inhibitory gene *Necdin* was decreased after inosine administration to premature white adipocytes (Extended Data Fig. 2j). In line, acute inosine treatment of isolated murine BAT induced significantly increases in oxygen consumption and lipolysis (Fig. 1j,k and Extended Data Fig. 2k).

Next, we studied the cellular receptors that mediate inosine effects in brown adipocytes. Given the stimulatory effect of inosine on cAMP and lipolysis, we focused on G_s_-coupled purinergic P1 receptors^22^. Incubation with the A_2A_-antagonist MSX-2 or the A_2B_-antagonist PSB603 significantly reduced the inosine-induced effect on lipolysis, measured as glycerol release, and the combination of both antagonists abrogated the inosine effect in murine brown adipocytes (Extended Data Fig. 2l).

## Inosine enhances energy expenditure

Given the stimulatory effect of inosine on thermogenic adipocytes and BAT explants, the effect of inosine on whole-body energy expenditure (EE) was studied by indirect calorimetry. Injection of inosine (100 µg kg^−1^) resulted in a significant increase in oxygen consumption in mice (Fig. 1l).

To study whether A_2A_ and A_2B_ mediate inosine effects in vivo, we used A_2A_-deficient (A2A-KO) mice and A_2B_-knockout (A2B-KO) mice. Wild-type (WT) and A2A- or A2B-KO mice were injected with inosine (100 µg kg^−1^) and oxygen consumption was monitored using metabolic cages. Inosine injections in WT mice resulted in increased (*P* < 0.05) oxygen consumption compared to vehicle-injected control animals, whereas the inosine effect was suppressed in A2A-KO or A2B-KO animals (Extended Data Fig. 2m,n). Taken together, our data show that inosine signals via the G_s_-coupled P1 receptors A_2A_ and A_2B_ in thermogenic adipocytes activating the cAMP/PKA/p38 pathway, thereby enhancing EE.

**Extended Data Fig. 3:**
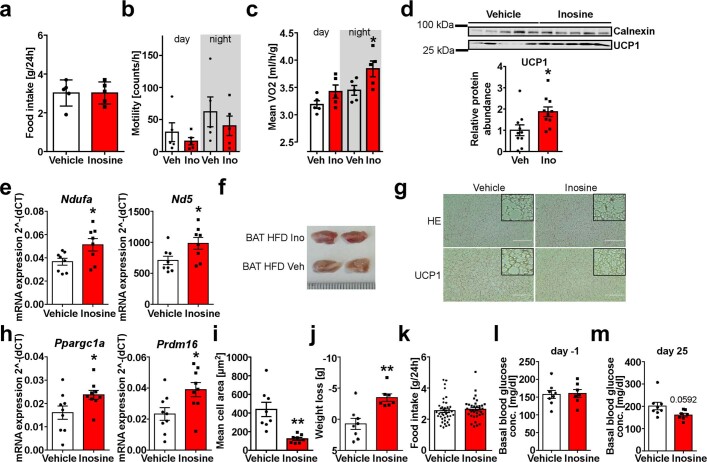
Inosine-mediated effects *in vivo*. **a–i**, Inosine administration via micro-osmotic pumps and HFD for 28 days in C57Bl6/J mice: **a**, Food intake (n = 5), **b** Motility (n = 5), **c**, Oxygen consumption at 23 °C (n = 5), **d**, Representative Western Blot and quantification of UCP1 expression in BAT (n = 10). Calnexin serves as a loading control. For gel source data see Supplementary Information. **e**, Expression of *Ndufa* and *Nd5* in inosine treated and control BAT (n = 8), **f**, Representative macroscopic pictures of BAT after 28 days of treatment and high fat diet (HFD), **g**, Representative haematoxylin/eosin (HE) and UCP1 staining of BAT sections (n = 3). Scale bar refers to 100 µm, 4-fold magnification of the picture in the upper right corner. **h**, Expression of *Ppargc1a* and *Prdm16* in WATi after inosine treatment (n = 9), **i**, Mean cell area of WATi (n = 8). **j–m** Mice were fed a HFD for 12 weeks, followed by daily injections (s.c.) with either vehicle (0.9% NaCl) or inosine (1 mg/kg) for 26 days during HFD (Vehicle n = 8, Inosine: n = 7): **j**, Weight loss (after 26 days). **k**, Food intake (5 measurements per mouse). **l–m**, Blood glucose concentrations at day -1 (**l**) and day 25 (**m**). For all: *p < 0.05, **p < 0.01, ***p < 0.005, ****p < 0.001. For exact p-values see Source Data. Data are represented as mean ± s.e.m. Two-tailed t-test was applied for **a–e**, **h**, **i**. Source data

To study whether inosine treatment might have beneficial effects during diet-induced obesity (DIO), micro-osmotic pumps were implanted in mice receiving a high fat diet (HFD) to apply inosine over 4 weeks. Mice on HFD treated with inosine gained significantly less weight in comparison to the vehicle-treated controls (Fig. 1m), while food intake and motility were not affected (Extended Data Fig. 3a,b). Inosine-treated mice on HFD consumed more oxygen (Fig. 1n and Extended Data Fig. 3c) and the maximal thermogenic capacity of brown/beige fat after acute cold exposure (4 °C) was significantly increased (Fig. 1o). Inosine-treated mice on HFD expressed significantly higher amounts of UCP1 mRNA and protein in BAT (Fig. 1p and Extended Data Fig. 3d). Moreover, expression of mitochondrial markers (*Ndufa* and *Nd5*) was increased in BAT after inosine treatment (Extended Data Fig. 3e). Macroscopically, BAT depots of mice receiving inosine had a more brownish appearance (Extended Data Fig. 3f). In addition, histological analysis of BAT sections demonstrated smaller lipid droplets in inosine-treated mice (Extended Data Fig. 3g). Analysis of inguinal white adipose tissue (WATi), the white depot with the highest ‘browning’ capacity^23^, showed significantly elevated expression of *Ucp1*, *Ppargc1a* and *Prdm16* (Fig. 1q and Extended Data Fig. 3h). Histological analysis showed more multilocular cells and decreased cell size in WATi of inosine-treated mice together with enhanced UCP1 staining compared to vehicle-treated mice (Fig. 1r and Extended Data Fig. 3i). These data show that inosine is a new activator of BAT-mediated EE and induces browning of WATi, thereby counteracting DIO.

To investigate the therapeutic potential of inosine, DIO mice were daily injected with either vehicle or inosine for 26 days and fed a HFD in parallel. We observed significant reductions in body weight of the inosine-injected mice compared to vehicle from day 7 onwards (Fig. 1s). As a consequence, the total weight loss after treatment was significantly higher in the inosine treatment group (Extended Data Fig. 3j). Inosine-injected mice showed significantly reduced fat mass, whereas body composition of the vehicle-treated mice did not change significantly (Fig. 1t). No difference in food intake was observed between the two groups (Extended Data Fig. 3k). Fasting blood glucose concentrations were not statistically different on the day before start of dosing (Extended Data Fig. 3l). By contrast, after 25 days of inosine injections and continuing HFD feeding, we observed a strong trend for reduced fasting blood glucose levels (*P* = 0.0592) (Extended Data Fig. 3m). In summary, these data further indicate that inosine counteracts obesity and has therapeutic potential also in established obesity.

## Regulation of inosine levels by ENT1

**Fig. 2:**
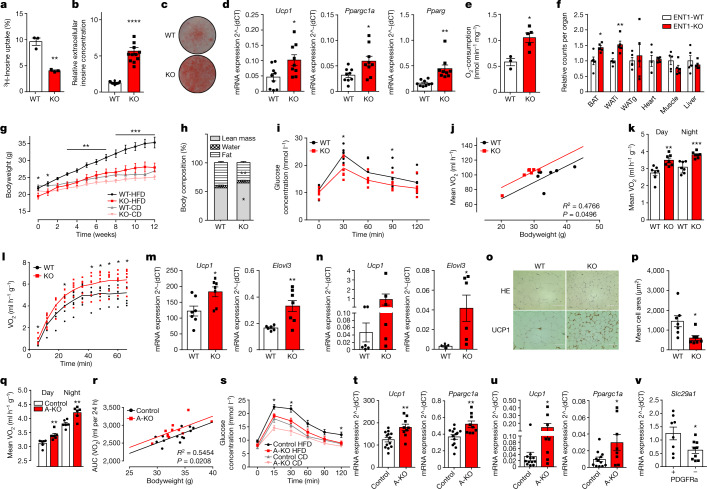
Role of ENT1 in adipose tissue metabolism. **a**–**d**, Analysis of WT and ENT1^−/−^ (KO) murine brown adipocytes. **a**, ^3^H-inosine uptake (*n* = 3). **b**, Extracellular inosine concentrations (*n* = 12). **c**, Representative Oil Red O staining. **d**, Expression of *Ucp1*, *Ppargc1a* and *Pparg* (*n* = 9). **e**, Oxygen consumption of WT and ENT1-KO BAT (*n* = 3–5). **f**, Lipid uptake (^14^C-triolein) in indicated organs of WT and ENT1-KO mice (*n* = 5, multiple *t*-tests). **g**, Body weight of WT and ENT1-KO mice during control diet (CD) or HFD (*n* = 7) (summarized *P* values of HFD groups are shown), **h**–**p**, Twelve-week HFD in WT and ENT1-KO mice. **h**, Body composition (*n* = 7). **i**, Glucose tolerance test (*n* = 7). **j**, ANCOVA of oxygen consumption/body weight (*n* = 7). **k**, Mean oxygen consumption over 24 h at 23 °C (*n* = 7). **l**, Oxygen consumption at 4 °C (*n* = 7). **m**, Expression of *Ucp1* and *Elovl3* in BAT (*n* = 6–7). **n**, Expression of *Ucp1* and *Elovl3* in WATi (*n* = 5–6). **o**, Representative haematoxylin and eosin and UCP1 staining of WATi (HE *n* = 7, UCP1 *n* = 3, scale bar, 100 µm). **p**, Mean adipocyte area of WATi (*n* = 7). **q**–**u**, Analysis of ENT1-floxed-AdiponectinCre mice. **q**, Oxygen consumption of 8-week old male mice (*n* = 6). **r**, ANCOVA of AUC of oxygen consumption/body weight after 12 weeks of HFD (13 control and 11 ENT1-A-KO mice were analysed). **s**, Glucose tolerance after 12 weeks of HFD or CD (HFD 13 control and ten ENT1-A-KO mice, CD *n* = 6) (summarized *P* values of the HFD groups are shown). **t**,**u**, Thermogenic marker expression in (**t**) BAT (13 control and nine ENT1-A-KO mice). **u**, WATi (12 control and eight ENT1-A-KO mice) after 12 weeks of HFD. **v**, *Slc29a1* expression in PDGFRα-positive and -negative stromal vascular fraction cells of WATi (*n* = 8–9). For all: *P < 0.05, **P < 0.01, ***P < 0.005, ****P < 0.001. For exact *P* values see source data. Data are represented as mean ± s.e.m. Two-tailed *t*-test was applied except for **j**,**r** (ANCOVA, non-linear fit) and **g**,**s** (one-way ANOVA with Tukey’s post hoc test). Source data

**Extended Data Fig. 4:**
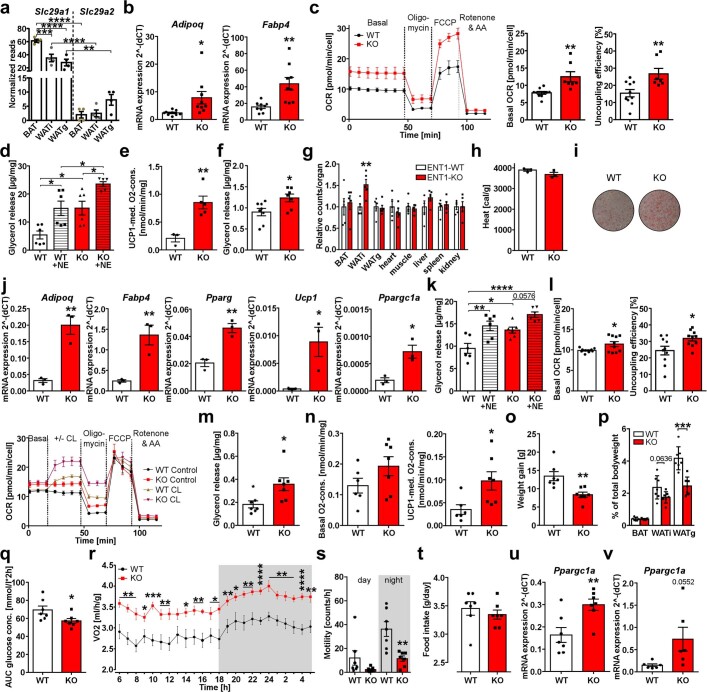
Global knockout of ENT1. **a**, RNA-sequencing analysis of *Slc29a1* and *Slc29a2* expression in adipocytes isolated from BAT, WATi, and WATg (n = 4). **b**, Expression of *Adipoq* and *Fabp4* in WT and ENT1 KO murine brown adipocytes (n = 9). **c**, Oxygen consumption of murine WT and ENT1-KO brown adipocytes (n = 7-10). **d**, Lipolysis of WT and ENT1-KO murine brown adipocytes stimulated with and without NE (1 µM) (n = 6). **e,f**, Analysis of WT and ENT1-KO BAT explants: UCP1-dependent respiration (n = 3-5) (**e**) and lipolysis (n = 7-8) (**f**). **g**, ^3^H-deoxyglucose (DOG) uptake into indicated organs of WT and KO mice (n = 5). **h**, Energy content of feces from WT and ENT1-KO mice (n = 3). **i**, Lipid droplet accumulation of WT and KO murine white adipocytes (n = 3). **j–l**, Analysis of WT and ENT1-KO murine white adipocytes: thermogenic and adipogenic marker expression (n = 3) (**j**), Lipolysis (n = 6) (**k**), Time course and statistical analyses of oxygen consumption (n = 9-10, for basal OCR all 4 groups and for uncoupling efficiency the 2 control groups were calculated) (**l**). **m**,**n**, Lipolysis (n = 7) (**m**) and oxygen consumption (n = 6-7) (**n**) of WT and ENT1-KO WATi explants. **o–v**, Twelve weeks of HFD in WT and ENT1-KO mice: Weight gain (n = 7) (**o**), Adipose tissue weight (n = 7) (**p**), AUC of glucose concentration (n = 7) (**q**), whole-body oxygen consumption at 23 °C (n = 7) (**r**), motility (n = 7) (**s**), food intake (n = 7) (**t**), *Ppargc1a* expression in BAT (n = 7) (**u**) and WAT (n = 6) (**v**). For all: *p < 0.05, **p < 0.01, ***p < 0.005, ****p < 0.001. For exact p-values see Source Data. Data are represented as mean ± s.e.m. Two-tailed t-test was applied except for **a**, **d** and **k** (One-way ANOVA with Tukey’s post hoc test). Source data

To address the question how extracellular inosine levels are regulated by adipocytes, we focused on transporters/channels that shuttle purines across the cell membrane. Whereas Pannexin1 channels have been shown to be important for nucleotide/ATP release during apoptosis^14^, equilibrative nucleoside transporters 1 and 2 (ENT1 and 2, respectively) can transport nucleosides such as inosine^24^. ENT1/*Slc29a1* has recently been shown to be a new marker for brown adipocytes^13^. As not much is known about the function of ENTs in adipocytes, *Slc29a1* and *Slc29a2* (ENT1 and 2 encoding genes, respectively) expression was analysed in primary adipocytes isolated from BAT, WATi and gonadal WAT (WATg). *Slc29a1* was by far the most highly expressed ENT-gene in adipocytes (Extended Data Fig. 4a) with brown adipocytes expressing significantly higher amounts of ENT1 than white adipocytes (Extended Data Fig. 4a). To study whether ENT1 is involved in inosine shuttling, brown adipocytes were isolated from ENT1-deficient (ENT1^−/−^) mice and uptake of ^3^H-labelled inosine was measured: ENT1^−/−^ brown adipocytes incorporated significantly less ^3^H-inosine from the cell culture supernatant than WT control cells (Fig. 2a) and subsequently more inosine accumulated in the supernatant of the ENT1^−/−^ brown adipocytes (Fig. 2b).

The analysis of the functional role of ENT1 showed an enhanced adipogenic and thermogenic differentiation with increased lipid droplet formation (Fig. 2c), enhanced expression of the thermogenic genes *Ucp1* and *Ppargc1a* as well as of adipogenic markers *Adipoq, Fabp4* and *Pparg* in ENT1^−/−^ brown adipocytes (Fig. 2d and Extended Data Fig. 4b). The enhanced differentiation of ENT1^−/−^ adipocytes is in accordance with the effects of apoptotic supernatant and/or inosine treatment (Fig. 1e,f). Moreover, oxygen consumption and lipolysis were significantly increased in ENT1^−/−^ brown adipocytes compared to WT cells (Extended Data Fig. 4c,d). Analysis of isolated tissues showed significantly increased basal and UCP1-mediated oxygen consumption in ENT1^−/−^ compared to WT BAT (Fig. 2e and Extended Data Fig. 4e). In line, the ex vivo lipolysis rate of ENT1^−/−^ BAT was also significantly higher compared to WT tissue explants (Extended Data Fig. 4f). These data indicate that loss of ENT1 increases inosine concentrations, thereby resulting in enhanced differentiation and thermogenic capacity as well as higher activation of brown adipocytes and BAT.

## ENT1 regulates EE

To analyse energy uptake into tissues in vivo, radioactive labelled glucose (^3^H-DOG) and fatty acids (^14^C-triolein) were administered to ENT1^−/−^ and WT mice. ^14^C-Triolein uptake was increased in BAT and WATi of ENT1^−/−^ animals (Fig. 2f). Moreover, glucose uptake was significantly increased in WATi (Extended Data Fig. 4g). Energy content of faeces was not significantly different between the two genotypes (Extended Data Fig. 4h). Adipogenesis was enhanced in ENT1^−/−^ white adipocytes as demonstrated by a higher number of lipid droplets as well as increased expression of adipogenic markers *Adipoq*, *Fabp4* and *Pparg* (Extended Data Fig. 4i,j). The expression of the thermogenic genes *Ucp1* and *Ppargc1a* was significantly increased in ENT1^−/−^ cells indicating enhanced browning of white adipocytes (Extended Data Fig. 4j). As a consequence, basal and NE-induced lipolysis as well as basal and uncoupled respiration was enhanced in ENT1^−/−^ white adipocytes compared to WT cells (Extended Data Fig. 4k,l). Analysis of WATi explants showed significantly increased lipolysis and UCP1-mediated respiration of ENT1^−/−^ WATi samples (Extended Data Fig. 4m,n). Basal respiration was increased by 49%, albeit not significantly (Extended Data Fig. 4n). These data indicate that ENT1 plays a major role in energy uptake in thermogenic adipose tissue and WAT browning.

## Loss of ENT1 protects against obesity

Next the effect of ENT1-deficiency on DIO was studied. Overall, ENT1^−/−^ mice gained significantly less weight during HFD compared to WT animals (Fig. 2g and Extended Data Fig. 4o) and had significantly less fat mass of (Fig. 2h) with WATi and WATg depots being reduced by 26 and 41%, respectively (Extended Data Fig. 4p). In addition, ENT1^−/−^ mice also showed significantly improved glucose tolerance after HFD (Fig. 2i and Extended Data Fig. 4q). ENT1^−/−^ mice dissipated significantly more energy compared to WT mice (Fig. 2j,k and Extended Data Fig. 4r). Motility of ENT1^−/−^ mice was reduced during the night cycle (Extended Data Fig. 4s), while food intake was not different between the genotypes (Extended Data Fig. 4t). Upon cold exposure, the total thermogenic capacity of ENT1^−/−^ mice, as well as thermogenic marker gene expression in ENT1^−/−^ BAT and WATi were significantly increased (Fig. 2l–n and Extended Data Fig. 4u,v). Histological analyses showed increased expression of UCP1 (Fig. 2o) and significantly decreased adipocyte size in ENT1^−/−^ WATi (Fig. 2p).

Taken together, ENT1^−/−^ mice show an increased BAT function and browning of WATi and are resistant to DIO.

## Adipose-specific knockdown of ENT1

**Extended Data Fig. 5:**
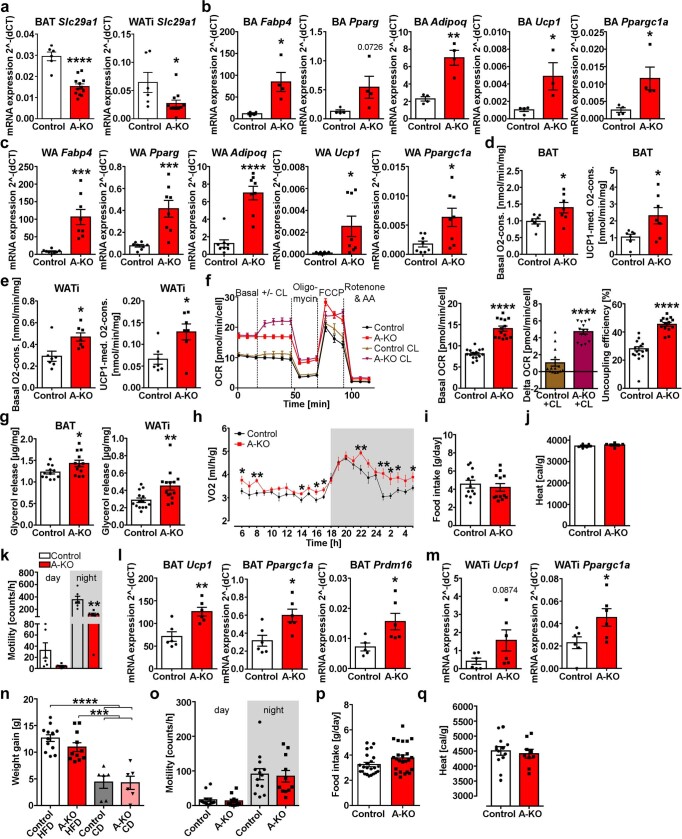
Adipocyte-specific deletion of ENT1. **a**, Expression of *Slc29a1* in BAT and WATi of ENT1-A-KO mice (6 control and 11-12 ENT1 A-KO mice were analyzed). **b**,**c**, Expression of adipogenic and thermogenic markers in control and ENT1-A-KO murine BA (n = 3-4) (**b**) and white adipocytes (WA) (n = 8) (**c**). **d**,**e**, *Ex vivo* oxygen consumption of control or ENT1-A-KO BAT (n = 7) (**d**) or WATi (n = 7) (**e**). **f**, Mitochondrial respiration of control or ENT1-A-KO WA (n = 14-15, for basal OCR all 4 groups for delta OCR the 2 CL groups and for uncoupling efficiency the 2 control groups were calculated). **g**, *Ex vivo* lipolysis of control or ENT1-A-KO BAT(n = 12) and WATi (n = 14). **h–m**, Characterization of 8-week-old male ENT1-A-KO mice (chow diet): whole-body oxygen consumption at 23 °C (n = 6) (**h**), food intake (6 mice were analyzed, 2 measurements per mouse) (**i**), energy content of feces (n = 6) (**j**), motility (n = 6) (**k**), thermogenic marker expression in BAT (n = 5-6) (**l**) and WATi (n = 6) (**m**). **n**, Weight gain of control and ENT1-A-KO mice on CD or HFD (13 control and 11 ENT1-A-KO mice on HFD and 6 mice per genotype on CD were analyzed). **o–q**, Analysis of control and ENT1-A-KO mice after HFD: motility (n = 11-13) (**o**), food intake (13 control and 11 ENT1-A-KO mice were analyzed, 1-3 measurements per mouse) (**p**) and energy content of feces (n = 9-13) (**q**). For all: *p < 0.05, **p < 0.01, ***p < 0.005, ****p < 0.001. For exact p-values see Source Data. Data are represented as mean ± s.e.m. Two-tailed t-test was applied except for **n** (One-way ANOVA with Tukey’s post hoc test). Source data

Given the broad expression pattern of ENT1, different cell types could be involved in the uptake of inosine. Therefore, we generated an adipose tissue-specific knockdown (A-KO) mouse model by crossing *Slc29a1*-floxed (ENT1fl) mice with adiponectin promoter-Cre mice (ApnCre) resulting in a 48 and 57% knockdown of ENT1 in BAT and WATi, respectively (Extended Data Fig. 5a). Significantly higher adipogenic (*Fabp4*, *Pparg*, *Adipoq*) and thermogenic (*Ucp1*, *Ppargc1a*) marker expression (Extended Data Fig. 5b,c), enhanced EE (Extended Data Fig. 5d–f) and lipolysis (Extended Data Fig. 5g) was observed in primary brown and white adipocytes as well as in BAT and WATi explants of A-KO mice, respectively. Whole-body EE of ENT1-A-KO mice was significantly increased compared to ENTfl littermates (Fig. 2q and Extended Data Fig. 5h). Food intake and the amount of energy secreted via faeces were not changed (Extended Data Fig. 5i,j), while the ENT1-A-KO animals moved less (Extended Data Fig. 5k). Similar to ENT1^−/−^ mice, significantly elevated expression of thermogenic genes (*Ucp1*, *Ppargc1a*, *Prdm16*) was observed in ENT1-A-KO BAT (Extended Data Fig. 5l). *Ucp1* and *Ppargc1a* expression was also increased in ENT1-A-KO WATi, albeit not significantly for *Ucp1* (Extended Data Fig. 5m).

ENT1-A-KO mice gained 13% less weight during 12 weeks of HFD compared to control littermates, albeit this difference was not significant (Extended Data Fig. 5n). ENT1-A-KO mice showed increased EE/oxygen consumption after 12 weeks of HFD compared to control litters (Fig. 2r). A-KO mice challenged with a HFD showed a significantly improved glucose tolerance in comparison to control animals (Fig. 2s). Motility, food intake and intestinal energy absorption were not changed between the two genotypes (Extended Data Fig. 5o–q). Expression of thermogenic genes *Ucp1* and *Ppargc1a* was increased in BAT and WATi of ENT1-A-KO after HFD compared to control mice (Fig. 2t,u). To further study the role of ENT1 in browning of adipocytes, we focused on PDGFRα expressing progenitor cells, which have been shown to differentiate towards beige/brite adipocytes^25^. *Slc29a1* expression was significantly higher in PDGFRα-positive stromal vascular fraction cells of WATi as compared to PDGFRα-negative cells (Fig. 2v).

Taken together, adipose tissue-specific knockdown of ENT1 recapitulates the phenotype of global ENT1^−/−^ mice showing that ENT1 in adipocytes plays a major role in regulation of thermogenesis and whole-body EE.

## Pharmacological inhibition of ENTs

**Extended Data Fig. 6:**
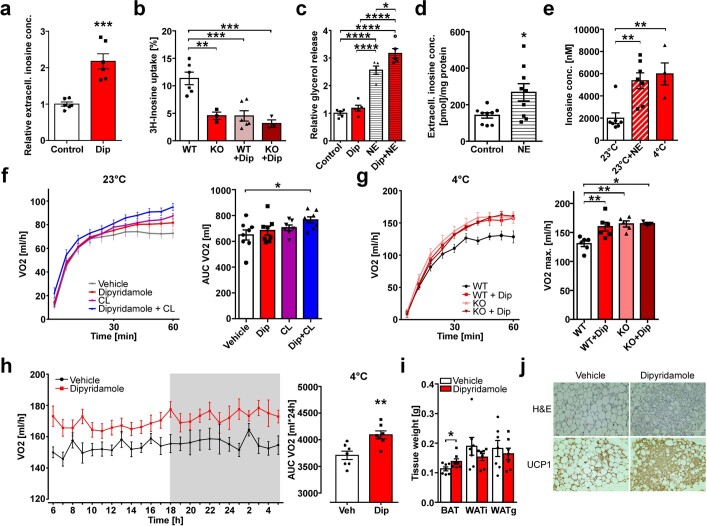
Effects of ENT1-inhibition by dipyridamole on adipose tissue. **a**, Extracellular inosine concentrations of murine BA treated with and without dipyridamole (Dip) (n = 6). **b**, ^3^H-Inosine uptake in WT and ENT1 KO murine BA treated with and without dipyridamole (Dip) (n = 3-6). **c**, Lipolysis of murine BA treated with and without dipyridamole (1 µM) in presence and absence of NE (1 µM) (n = 5). **d**, Extracellular inosine levels of murine BA treated with and without NE (n = 9). **e**, Tissue inosine concentrations of BAT dialysates from mice housed at 23 °C treated with and without NE, or from cold exposed mice (4 °C) (n = 4-7). **f**, Oxygen consumption at 23 °C of C57Bl6/J mice after injection of vehicle, the β3-adrenergic agonist CL316,243 (0.3 µg/kg) in the presence or absence of dipyridamole (1 mg/kg) (n = 7-8) (left graph: timecourse, right graph: AUC). **g**, Oxygen consumption at 4 °C in WT and ENT1-KO mice after injection of vehicle or dipyridamole (1 mg/kg) (Dip) (n = 3-6) (left graph: timecourse, right graph: V02 max.). **h–j**, Analysis of C57Bl6/J mice after 7 days of cold exposure (4 °C) with and without daily dipyridamole injection (1 mg/kg): h, Oxygen consumption (n = 7), i, Weights of adipose tissues (n = 7), **j**, Representative HE and UCP1 staining of WATi sections (n = 3). For all: *p < 0.05, **p < 0.01, ***p < 0.005, ****p < 0.001. For exact p-values see Source Data. Data are represented as mean ± s.e.m. Two-tailed t-test was applied for **a**, **d**, **h**, **i**, One-way ANOVA with Tukey’s post hoc test for **b**, **c**, **e**, **f**, **g**. Source data

Treatment of brown adipocytes with dipyridamole, an approved antiplatelet drug that blocks ENTs^26^, led to a significant increase of extracellular inosine (Extended Data Fig. 6a) and inhibited inosine uptake (Extended Data Fig. 6b). Although dipyridamole alone induced a 18% increase in lipolysis (Extended Data Fig. 6c), this effect was not significantly different from control indicating that inosine release first has to be triggered for pharmacological ENT1 inhibition to achieve significant effects. To study which stimuli might induce inosine accumulation in BAT apart from apoptosis, we reasoned that inosine might accumulate during physiological sympathetic activation of BAT, which has been shown to induce the release of the precursor of inosine, adenosine^27^. Indeed, activation of brown adipocytes with norepinephrine (NE) induced a significant increase in extracellular inosine (Extended Data Fig. 6d). To directly analyse inosine concentrations in BAT tissue, we established a microdialysis set-up and found that acute activation of BAT with NE or cold exposure of mice at 4 °C for 7 days, increased inosine amounts 2.7- and 3.0-fold (Extended Data Fig. 6e). These data show that BAT releases inosine also after physiological stimulation, thus constituting a so far unknown positive feed-forward loop that could contribute to sustained BAT activation after sympathetic activation of EE.

As brown adipocytes treated with dipyridamole together with NE showed a significant additive effect on NE-induced activation (lipolysis) (Extended Data Fig. 6c), we studied mice injected with dipyridamole in the presence and absence of the β3-adrenoceptor agonist CL 316243 (CL) using a concentration of CL 316243 (0.3 mg kg^−1^), which alone resulted in only a minor, non-significant induction of EE (Extended Data Fig. 6f). Whereas dipyridamole alone led to a non-significant increase of oxygen consumption, cotreatment of mice with dipyridamole and CL 316243 induced a significant increase of oxygen consumption compared to vehicle (Extended Data Fig. 6f). Similarly, acute injection of dipyridamole in cold-exposed mice induced a significant increase in oxygen consumption, comparable to the amounts observed in ENT1^−/−^ mice (Extended Data Fig. 6g).

To study whether ENT1 blockade has an effect on browning, we injected dipyridamole for 7 days during cold exposure (4 °C) in WT mice. Dipyridamole treatment led to a significant increase in oxygen consumption in mice compared to vehicle-injected littermates (Extended Data Fig. 6h). Mice injected with dipyridamole had significantly larger BAT mass compared to vehicle-treated mice (Extended Data Fig. 6i), while tissue weights of WATi and WATg were reduced by 19 and 10%, respectively (Extended Data Fig. 6i). Immunohistological analysis showed a smaller adipocyte surface area and more UCP1 positive beige cells in WATi depots in dipyridamole-treated mice (Extended Data Fig. 6j).

These data show that inhibition of ENTs synergistically increases EE from physiologically or pharmacologically activated BAT as well as enhances browning of WATi.

## Inosine activates human brown adipocytes

To study the role of inosine in human brown adipocytes (hBA), we isolated primary human preadipocytes from neck biopsies and differentiated them to mature brown adipocytes^27,28^.

**Fig. 3:**
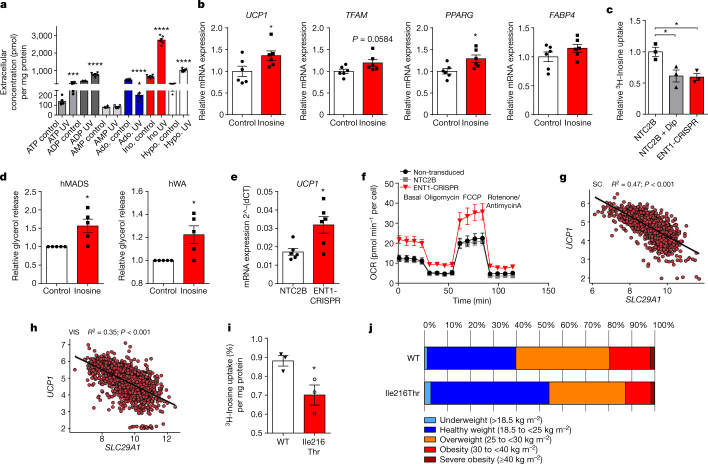
Regulation of human adipocytes by inosine and SLC29A1. **a**, Concentrations of purinergic molecules in the supernatant of human brown adipocytes after UV irradiation (*n* = 6). **b**, Expression of thermogenic (*UCP1, TFAM*) and adipogenic (*PPARG, FABP4*) marker genes in human brown adipocytes treated with and without inosine (*n* = 6). **c**, ^3^H-Inosine uptake in human brown adipocytes with (ENT1-CRISPR) and without (NTC2B) ENT1 knockdown treated with and without dipyridamole (Dip) (1 µM) (*n* = 3). **d**, Lipolysis of human beige (hMADS) and human white adipoctyes (hWA) treated with and without inosine (300 nM) (*n* = 5). **e**, UCP1 expression of hWA with (ENT1-CRISPR) and without (NTC2B) ENT1 knockdown (*n* = 6). **f**, Oxygen consumption rate (OCR) of hWA with (ENT1-CRISPR) and without (NTC2B) ENT1 knockdown (*n* = 4). **g**,**h**, Linear regression between SLC29A1 and UCP1 expression in human subcutaneous WAT (subcutaneous, SC) (**g**) (*n* = 1,476; *ρ* = −0.72) and human visceral WAT (visceral, VIS) (**h**) (*n* = 1,583; *ρ* = −0.61), *P* values were corrected for multiple inference using the Holm method. **i**, ^3^H-inosine uptake of HEK293T cells overexpressing either the ENT1-WT or Ile216Thr variant (*n* = 3). **j**, BMI of Ile216Thr variant carriers (Ile/Thr and Thr/Thr) compared to the Ile/Ile homozygous participants (n(Ile/Thr) = 72, n(Thr/Thr) = 822). For all: *P < 0.05, **P < 0.01, ***P < 0.005, ****P < 0.001. For exact *P* values see source data. Data are represented as mean ± s.e.m. A two-tailed *t*-test was applied for **a**,**b**,**d**,**e**,**i** and one-way ANOVA with Tukey’s post hoc test for **c**. Source data

**Extended Data Fig. 7:**
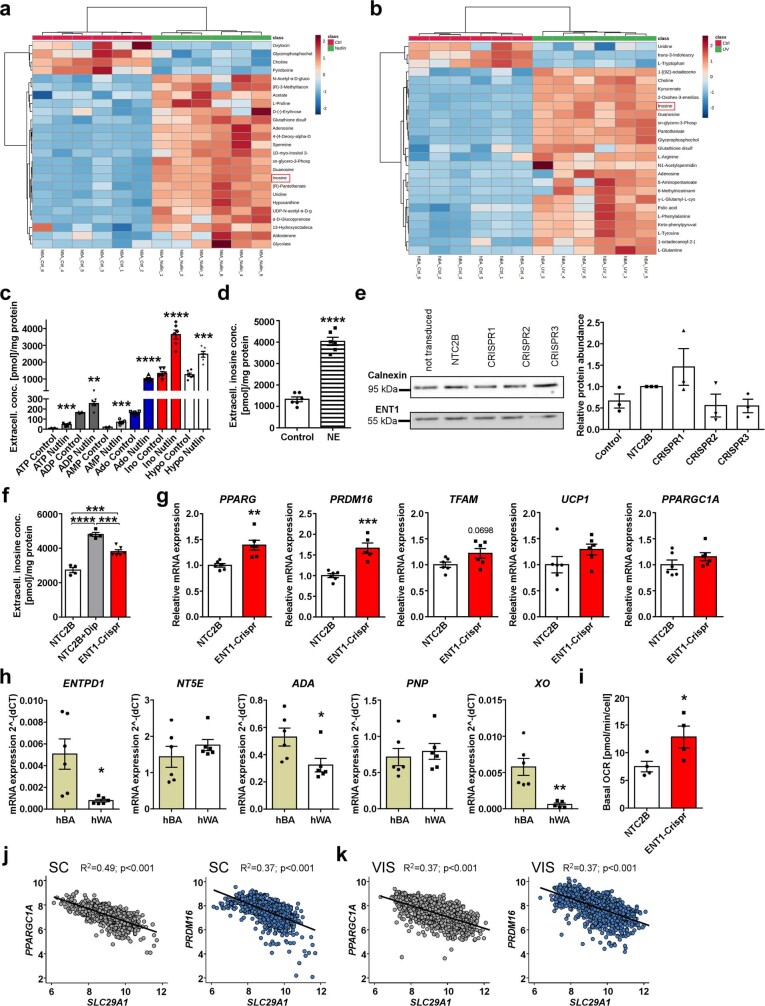
Inosine- and ENT1-mediated effects on human adipocytes. **a**, **b** Heatmap of top 25 secreted metabolites of untargeted metabolomics of human BA: (a) treated with and without nutlin-3 (b) irradiated with UV, upregulated metabolites are shown in red, downregulated metabolites are shown in blue (n = 6). **c**, Concentrations of purinergic molecules in the supernatant of human BA treated with and without nutlin-3 (n = 6). **d**, Extracellular inosine concentrations of human BA treated with and without NE (n = 6). **e**, Representative Western Blot and quantification for ENT1 knockdown in human BA induced by different lentiviral Crispr-Cas9 vectors (CRISPR1, 2, 3) in comparison to a control vector (NTC2B) and to not-transduced (Control) cells (n = 3). Calnexin serves as a loading control. For gel source data see Supplementary Information. **f**, extracellular inosine concentrations in human BA with (ENT1-Crispr) and without (NTC2B) ENT1 knockdown treated with and without Dipyridamole (Dip) (1µM) (n = 4-6). **g**, mRNA expression of adipogenic (*PPARG)* and thermogenic (*PRDM16, TFAM, UCP1, PPARGC1A*) marker genes in human BA with (ENT1-Crispr) and without (NTC2B) ENT1 knockdown (n = 5-6). **h**, Expression of the purinergic enzymes *ENTPD1, NT5E, ADA, PNP* and *XO* in human brown (hBA) and white (hWA) adipocytes (n = 5-6). **i**, Basal oxygen consumption rate (OCR) of hWA with (ENT1-Crispr) and without (NTC2B) ENT1 knockdown (n = 4). **j**, **k** Linear regression of *SLC29A1* and (**j**) *PPARGC1A* expression (n = 1,476; ρSpearman = −0.71) and *PRDM16* expression (n = 1,476; ρSpearman = −0.64) in human subcutaneous WAT (SC) and (**k**) *PPARGC1A* expression (n = 1,583; ρSpearman = −0.6) and *PRDM16* expression (n = 1,583; ρSpearman = −0.63) in human visceral WAT (VIS), p-values were corrected for multiple inference using the Holm method. For all: *p < 0.05, **p < 0.01, ***p < 0.005, ****p < 0.001. For exact p-values see Source Data. Data are represented as mean ± s.e.m. Two-tailed t-test was applied for **c**, **d**, **g**, **h**, **i**, One-way ANOVA with Tukey’s post hoc test for **a**, **b**, **e**, **f**.

Similar to our approach in murine brown adipocytes, we first set the experimental conditions to induce apoptosis by treatment with Nutlin-3 or UV light in hBA without disrupting the cell membrane. Untargeted metabolomics showed increased extracellular inosine concentrations for both apoptotic conditions (Nutlin-3 and UV irradiation) (Extended Data Fig. 7a,b). Targeted analysis of purinergic molecules further showed that extracellular inosine concentrations were significantly increased in human brown adipocytes after UV irradiation or Nutlin-3 treatment (Fig. 3a and Extended Data Fig. 7c). Inosine was the most abundant purine released by hBA under both apoptotic conditions (Fig. 3a and Extended Data Fig. 7c). Moreover, stimulation of hBA with NE resulted in significantly increased accumulation of extracellular inosine (Extended Data Fig. 7d).

Similar to murine brown adipocytes (Fig. 1h), treatment of hBA with inosine (300 nM) resulted in increased expression of the thermogenic genes *UCP1* and *TFAM* as well as the adipogenic differentiation markers *PPARG* and *FABP4* (Fig. 3b). To knockdown SLC29A1/ENT1 in hBA, CRISPR–Cas9 lentiviral vectors were used; one construct (CRISPR3) was identified mediating the highest knockdown of ENT1 and was subsequently used for all following ENT1-knockdown (ENT1-KD) experiments (Extended Data Fig. 7e). ENT1-KD adipocytes took up significantly less ^3^H-inosine compared to control cells (Fig. 3c). The reduction of inosine uptake observed in ENT1-KD cells was similar to the effect of dipyridamole in hBA (Fig. 3c). These findings were in line with increased extracellular inosine concentrations measured in dipyridamole-treated and ENT1-CRISPR transduced hBA (Extended Data Fig. 7f). Similar to murine ENT1^−/−^ brown adipocytes, mature ENT1-KD hBA showed significantly upregulated *PPARG* and *PRDM16* expression (Extended Data Fig. 7g), and expression of the thermogenic marker genes *TFAM*, *UCP1* and *PPARGC1A* were increased by 22, 29 and 15%, respectively, albeit not significantly (Extended Data Fig. 7g).

Next, we compared the expression levels of purinergic enzymes of human brown and white adipocytes and found that the ATP-degrading enzyme ENTPD1 and the xanthine oxidase were significantly higher expressed in hBA (Extended Data Fig. 7h). Adenosine deaminase, the enzyme converting adenosine to inosine, was also significantly higher expressed in hBA (Extended Data Fig. 7h).

To study the effect of inosine in human white adipocytes (hWA) and beige adipocytes, we used primary hWA and the hMADS cell line, respectively. In both hWA and hMADS, inosine significantly increased lipolysis (Fig. 3d). Knockdown of ENT1 in hWA resulted in increased *Ucp1* expression and oxygen consumption indicating browning of hWA after reduction of ENT1 function (Fig. 3e,f and Extended Data Fig. 7i).

Taken together, apoptotic hBA release inosine and inhibition or knockdown of ENT1 increases extracellular inosine levels. Modulation of inosine/ENT1 has a big impact on differentiation and function of human brown and beige adipocytes.

## SLC29A1 levels in human adipose tissue

Next, we analysed the correlation of *SLC29A1* expression with expression levels of thermogenic marker genes such as *UCP1, PPARGC1A* and *PRDM16* in human subcutaneous and visceral WAT in a large cohort (more than 1,400 participants). We found that expression of *SLC29A1*/ENT1 significantly negatively correlates with *UCP1* in human subcutaneous WAT (*n* = 1,476; Spearman's rho (*ρ*) was −0.72) and in human visceral WAT (*n* = 1,583; *ρ* = −0.61) (Fig. 3g,h). Moreover, there was a significant negative correlation of *SLC29A1* expression with *PPARGC1A* expression in visceral (*n* = 1,583; *ρ* = −0.6) and in subcutaneous WAT (*n* = 1,476; *ρ* = −0.71) (Extended Data Fig. 7j,k). *SLC29A1* levels also negatively correlated to *PRDM16* expression in both visceral (*n* = 1,583; *ρ* = −0.63) and in subcutaneous WAT (*n* = 1,476; *ρ* = −0.64) (Extended Data Fig. 7j,k). These data indicate that reduced *SLC29A1* expression, which in turn mediates increased extracellular inosine concentrations, correlates with higher expression of thermogenic genes in human WAT.

## ENT1-mutant associates with lower body mass index

Analysis of The Genome Aggregation Database (gnomAD) showed several variants in *SLC29A1* with missense mutations. The variant with the highest frequency (gnomAD allele frequency 0.026 in European, non-Finnish population with 0.03% homozygous carriers) was a single nucleotide polymorphism c.647T>C in *SLC29A1* (dbSNP rs45573936; chr6:g.44230625T>C (hg38)) leading to a p.Ile216Thr substitution (Ile216Thr). Overexpression of the Ile216Thr variant using lentiviral vectors resulted in significantly reduced ^3^H-inosine uptake as compared to overexpression of the WT variant (Fig. 3i). Thus, establishing that the Ile216Thr substitution is detrimental to the function of ENT1.

To address the potential clinical relevance of the Ile216Thr variant, we tested its association with body mass index (BMI) in an extensively phenotyped study cohort derived from a self-contained population of Sorbs in Germany^29^. According to the exclusion criteria—age below 18 years, pregnancy or lactation period, acute infections and diabetes—895 participants with available genotypes were included in the analyses. The frequency of the minor allele was 4% and the distribution of genotypes was in Hardy–Weinberg equilibrium (*P* > 0.05). The minor C allele was significantly associated with lower mean BMI (beta −0.98, 95% CI (−1.959, −0.005); *P* = 0.049 after adjusting for age and sex) (Fig. 3j). Most of all, participants with underweight or healthy weight were over-represented in the group of Thr variant carriers (Ile/Thr and Thr/Thr) compared with the Ile/Ile homozygous participants (55 versus 40%) (Fig. 3j). In line with this, the Thr variant carriers had 59% lower odds of obesity (BMI < 25 versus BMI > 30 kg m^−2^) compared with non-carriers (per-allele odds ratio (OR), 0.41; 95% CI, 0.17, 0.96; *P* = 0.04 after adjusting for age and sex, Fig. 3j).

## Discussion

**Extended Data Fig. 8:**
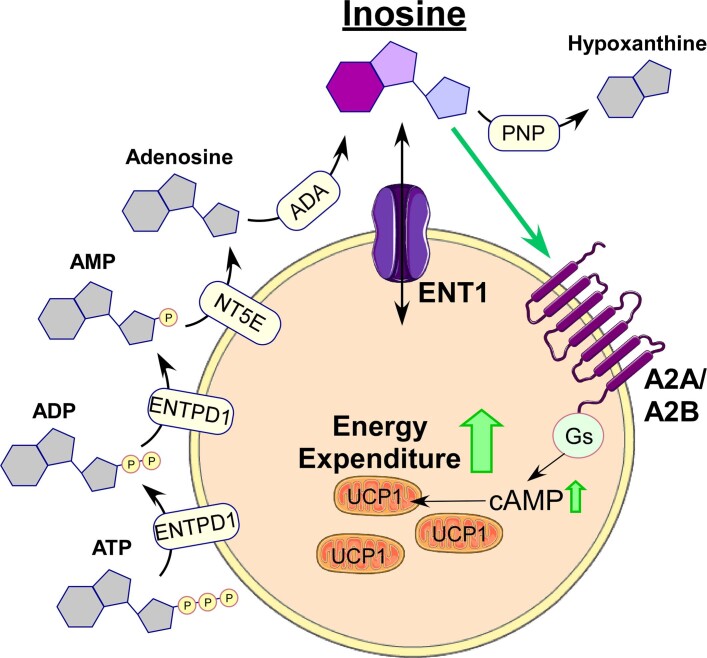
Schematic representation of inosine production, clearance and action in thermogenic adipocytes. ATP and ADP are degraded by ectonucleoside triphosphate diphosphohydrolase 1 (ENTPD1) to AMP, which in turn is metabolized to adenosine by ecto 5’-nucleotidase (NT5E). Inosine is generated by adenosine deaminase (ADA) or is released into the interstitial space via ENT1. Inosine signaling is terminated by either uptake of inosine into the cell by ENT1 or degradation by purine nucleoside phosphorylase (PNP). Extracellular inosine exerts its effects via the purinergic P1 receptors A_2A_ and A_2B_ resulting in G_s_ protein-induced cAMP production. Parts of the figure were drawn by using pictures from Servier Medical Art, provided by Servier, licensed under a Creative Commons Attribution 3.0 unported license (https://creativecommons.org/licenses/by/3.0/).

The central nervous system is generally believed to be the key regulator of thermogenic adipose tissue. However, a new concept has been emerging that focusses on local signals that control recruitment of brown and beige adipocytes^30,31^. Here, we identify inosine as a tissue messenger and para- or autocrine signalling molecule that is a main driver of EE from brown/beige adipocytes. Using untargeted metabolomics, we found that dying brown adipocytes release a complex pattern of nucleotides/nucleoside with rather low ATP concentrations. This may be explained by the concerted action of different enzymes that degrade ATP and generate a whole range of purine metabolites (Extended Data Fig. 8). AMP, inosine and hypoxanthine reached the highest concentration, but only inosine activated cAMP signalling and EE in brown adipocytes. Mechanistically, we found that in brown adipocytes inosine activates purinergic P1 receptors (A_2A_ and A_2B_), which belong to the family of G_s_-coupled G-protein-coupled receptors^22^. Inosine signalling activates PKA and the classical inducers of the thermogenic programme p38, SIK2, CRTC3 and CREB^21,20^. Moreover, our study identifies ENT1 as main regulator of nucleosides in brown adipocytes. Loss of ENT1 suppressed inosine uptake into adipocytes and thus inosine accumulates in the extracellular space. Although adenosine has been shown to acutely induce brown adipocyte activation^27,32^, it is rapidly degraded by adenosine deaminase to inosine, which has a longer half-life^33^.

We identified inosine in the context of apoptosis; however, this purine plays also a more general role in physiological activation of brown fat and inosine treatment enhances expression of the thermogenic programme; accordingly, loss of ENT1 enhances differentiation of brown adipocytes and induces browning of white adipocytes. Increasing inosine concentrations in vivo via inosine injection or ablation of ENT1 increases EE and protects mice against DIO.

In human adipocytes, inosine had similar positive effects as in murine cells. Apoptotic human brown adipocytes release inosine and knockdown of *ENT1* caused accumulation of inosine that induced browning of human white adipocytes and increased EE. In a large human cohort, we found that ENT1 inversely correlated with the expression of thermogenic markers in human adipose tissue. A missense mutation in *SLC29A1* (c.647T>C, p.Ile216Thr) is associated with decreased BMI and a reduced likelihood of obesity, further underlining the role of ENT1 in human metabolism and energy homeostasis.

BAT stimulation using adrenergic agonists is hampered by significant cardiovascular side-effects^34^. Thus, targeting ENT1/increasing inosine might be an alternative or synergistic approach for future anti-obesity therapies.

## Methods

### In vivo methods

#### Animals

C57BL/6J mice were obtained from Charles River. ENT1-KO and ENT1-floxed mice were kindly provided by H.K. Eltzschig. Generation of ENT1-null mice has been described by Choi et al.^35^. ENT1-floxed mice were bred with B6;FVB-Tg(Adipoq-cre)1Evdr/J (Jackson Laboratory, stock no. 010803) to obtain animals with homozygous loxP-flanked ENT1-alleles without Cre or hemizygous for Cre. A_2A_ knockout animals^36^ were purchased from The Jackson Laboratory (Strain C, 129-Adora2atm1fc/J). A_2B_-KO mice^37^ were provided by M. Idzko, Freiburg, Germany. All animal experiments have been approved by the local authorities including Landesamt für Natur, Umwelt und Verbraucherschutz, NRW, Germany, Behörde für Gesundheit und Verbraucherschutz Hamburg, Hamburg, Germany and Institutional Animal Care and Use Committee (protocol CBSD 21-013), San Diego, CA, USA.

Mice were housed in the respective animal facilities with a light and dark cycle of 12 h each and access to chow or HFD (as indicated) and water ad libitum at ambient room temperature and humidity.

#### DIO

Six-week-old male mice were fed with HFD (Ssniff, D12492) or control diet (Ssniff, D12450B) for 12 weeks and the body weight was monitored weekly.

#### Body composition analysis

Body composition was analysed using a Bruker Minispec LF50H.

#### Glucose tolerance test

Animals were fasted for 5 h and 8 µl g^−1^ body weight of glucose solution (0.25 g ml^−1^) were injected intraperitoneally (i.p.): glucose was measured before and at indicated time points postinjection. Tail vein was punctured and blood was analysed with Accu Check (Aviva Nano) analyser and dipsticks (Roche).

#### Metabolic characterization

For metabolic characterization of mice, oxygen consumption and motility were measured at 23 °C for 24 h, with a light and dark cycle of 12 h each, using the Phenomaster system (TSE Systems). During the measurements mice were single caged with access to food and water ad libitum. Acute cold exposure of mice was performed for 1–2 h at 4 °C, during the light cycle and without access to food and water.

#### Thermoneutrality experiments

Thermoneutrality experiments were performed in J. Heeren’s laboratory (Department of Biochemistry and Molecular Cell Biology, University Medical Center Hamburg-Eppendorf, Hamburg, Germany). The 10–13-week old, male C57BL/6J mice were either housed at 22 °C or at thermoneutrality (30 °C) for 3 or 7 days before organ isolation.

#### Acute injections of inosine

Eight-week old, male WT mice (C57BL/6 background), A2A-KO and A2B-KO mice were injected with inosine (i.p., 100 µg kg^−1^, dissolved in NaCl 0.9%) (Sigma-Aldrich, I4125) and oxygen consumption at 23 °C was monitored using metabolic cages (Phenomaster, TSE Systems).

#### Acute injections of dipyridamole

Eight-week old, male WT mice (C57BL/6 background) were injected (i.p.) with dipyridamole (1 mg kg^−1^) (Sigma-Aldrich, D9766) and/or CL316243 (0.3 µg kg^−1^) (Tocris, 1499) dissolved in the vehicle (50% DMSO in NaCl 0.9%) or vehicle. Oxygen consumption at 23 or 4 °C was monitored using metabolic cages (Phenomaster, TSE Systems).

#### Implantation of micro-osmotic pumps

Micro-osmotic pumps (alzet, model 1004, catalogue no. 0009922) were implanted subcutaneously into 8-week-old, male C57Bl6/J mice, under anaesthesia (3.5% isoflurane), following the manufacturer’s instructions. Subsequently, inosine (0.11 µl h^−1^ of 3.4 mM solution, 2.4 µg per day) (Sigma-Aldrich, order no. I4125) or vehicle (NaCl 0.9%) was permanently released for 28 days. During this period, mice were either fed a control diet or HFD and the body weight was monitored. Indirect calorimetry measurements were performed from day 22 until day 27. After 28 days, the mice were euthanized and the organs were isolated for analysis.

#### Dipyridamole injections and 7 days of cold exposure

Eight-week old, male WT mice (C57BL/6 background) were kept in metabolic cages (Phenomaster, TSE Systems) with access to chow diet and water ad libitum. All animals were acclimatized for 3 days at 16 °C and subsequently housed at 4 °C for 7 days. Dipyridamole (1 mg kg^−1^) (Sigma-Aldrich, D9766) dissolved in the vehicle (50% DMSO in PBS) or vehicle were daily injected subcutaneously between the shoulder blades.

#### Inosine injections in obese mice

The DIO-mice injection experiments were performed at Crown Bioscience. The 18-week-old, male mice, which were DIO after 12 weeks of HFD (Sniff, DIO Diet D12492) (Jax strain 380050), were daily injected (s.c.) with either vehicle (0.9% NaCl) or 1 mg kg^−1^ inosine (Sigma-Aldrich, order no. I4125) and continued being fed a HFD (Sniff, DIO Diet D12492). Fasting blood glucose concentrations (after 6 h) and EchoMRI (quantitative nuclear magnetic resonance) body composition measurements were performed 1 day before start of daily dosing. A further measurement of fasting blood glucose concentrations was performed after 25 days of dosing and further EchoMRI (quantitative nuclear magnetic resonance) body composition measurements were performed after 26 days of dosing. Body weight and daily food intake were monitored one to six times per week. Animals were euthanized after 26 days of dosing and organs and blood were isolated.

#### Radioactive labelled glucose and fat uptake

Metabolic tracer studies in 8-week-old male ENT1-WT and -KO mice were performed at the Department of Biochemistry and Molecular Cell Biology, University Medical Center Hamburg-Eppendorf, Hamburg, Germany. A lipid emulsion, labelled with ^14^C-triolein (0.15 MBq per kg) and ^3^H-DOG (0.72 MBq per kg), was administered to the mice by oral gavage. Mice were euthanized 2 h post gavage application and organs were dissected and homogenized. Subsequently, radioactivity of respective solubilized organs was measured by liquid scintillation counting.

#### Bomb calorimetry of faeces

A 6725 Semimicro Bomb Calorimeter (Parr) was used to measure the heat produced by the combustion of murine faeces samples. 0.02–0.04 g of faeces were placed in an Inconel dish of a 1109A Semimicro Oxygen Bomb (Parr). Afterwards, a 10-cm Ni-Cr loop was installed causing the wire to touch the samples. Finally, the bomb was closed, saturated with oxygen and placed into a stainless steel air can containing 400 ml of dd-H_2_O. After preparation of electrical connections for the firing circuit, the samples were burned and the resulting rise in temperature was measured enabling calculation of heat.

### Ex vivo analysis

#### Microdialysis

Fat tissues were isolated from 8-week-old male C57/Bl6J mice, which were housed for 7 days either at 23 or 4 °C. Next, microdialysis membranes (CMA 30 Linear MD Probe, catalogue no. 8010460) were implanted. Tissues were placed in oxygen saturated buffer and perfused with perfusion fluid (M Dialysis AB, catalogue no. P000034) at a flow of 1 µl min^−1^ using a syringe pump (CMA). After 2 h the flow through of 30 min was collected. Inosine concentrations of the dialysate were measured with a ultra-high-performance liquid chromatogtaphy with tunable UV (UPLC-TUV) system (Waters).

#### Immunohistochemistry

For UCP1 staining, 5-μm paraffin-embedded BAT and WAT sections were blocked with 2.5% normal goat serum–PBST (phosphate-buffered saline + 0.1% Tween-20) for 1 h at room temperature. Primary antibody (UCP1, custom made; 1:250) was applied overnight at 4 °C. After washing three times with PBST, secondary antibody against rabbit (SignalStain Boost IHC, Cell Signaling, catalogue no. 8114S, ready to use or undiluted) was applied for 1 h at room temperature and developed with DAB Kit (Vector Laboratories) according to the manufacturer’s instructions. Standard haematoxylin and eosin staining was performed on 5-μm paraffin-embedded BAT and WAT sections. Pictures of stained slides were taken with EVOS FL Color Imaging System. Quantification of cell size in haematoxylin and eosin-stained tissue sections was analysed and calculated using ImageJ2 software. Contrast and brightness of the pictures were adjusted using Canvas 11 software.

#### Measurement of endogenous respiration

BAT and WATi was treated as indicated (vehicle or 300 nM inosine) 15 min before oxygraphic measurements (Oxygraph 2K, Oroboros Instruments). Samples were transferred to the oxygraph chamber containing 2 ml incubation medium (0.5 mM EGTA, 3 mM MgCl_2_ 6H_2_O, 60 mM K-lactobionate, 20 mM taurine, 10 mM KH_2_PO_4_, 20 mM HEPES, 110 mM sucrose and 1 g l^−1^ bovine serum albumin (BSA), pH 7.1). Ex vivo respiration levels were recorded when reaching a steady state followed by addition of substrates (state 1, endogenous; state 2, substrates, succinate; state 3, GDP; state 4, sodiumazide; uncoupled, FCCP (a mitochondrial uncoupler)). Respiration rates were normalized to wet tissue weight.

### In vitro methods

#### Primary human and murine adipocyte culture

Stromal vascular fraction cells from human supraclavicular adipose tissue biopsies and mouse intrascapular BAT were isolated and differentiated as described previously^38,39^. hMADS were provided by the laboratory of C. Dani (University of Nice Sophia Antipolis) and differentiated as described^40^. hWA were obtained from Lonza and differentiated according to the manufacturer’s instructions. Murine white adipocytes were differentiated as described^41^.

#### Isolation of mature adipocytes, endothelial cells and tissue-resident macrophages from mice housed at 22 or 30 °C for 3 days

Cell sorting of interscapular BAT was performed as described previously^42^. In brief, pooled BAT was digested in PBS containing 10 mM CaCl_2_, 2.4 units per ml dispase (17105-041, Gibco) and 1.5 units per ml collagenase D (11088882001, Roche) (45 min, 37 °C). The cell suspension was filtered through a 100-µm cell strainer and centrifuged for 5 min (600*g*, 4 °C). The supernatant was collected as adipocyte fraction. After resuspension of the pellet, the remaining cells were passed through a cell strainer (40 µm). CD11b^+^ cells were isolated with CD11b MicroBeads (130-049-601, Miltenyi; 10 µl beads per 10^7^ cells) using magnetic columns (Miltenyi, 130-042-401). After centrifugation of the flow through, the pellet was resuspended and CD31 MicroBeads were added (130-097-418, Miltenyi; 10 µl beads per 10^7^ cells). After incubation, CD31^+^ cells were pulled out with magnetic columns (Miltenyi, 130-042-401). For RNA isolation, pellets with cell fractions were resuspended in Trizol reagent.

#### Isolation of BAT-derived fibroblasts

BATs were minced and digested in digestion buffer (DMEM containing 0.5% BSA and 1.5 mg ml^−1^ Collagenase II (catalogue no. CLS2)). After digestion, all tissue debris were removed by filtration using a 100-µm nylon mesh (Merc Milipore, NY1H00010). Samples were centrifuged and the pellet was resuspended in DMEM (+10% FBS, +1% P/S). After 2 h the medium was changed to fresh DMEM (+10% FBS, +1%Pen/Strep) (differential attachment). Cells were grown to 80–90% confluence, 50 µl of Dynabeads (Creative Diagnostics, WHM-S016) were added and cells were incubated at 37 °C for 30 min. Afterwards, the cells were detached using trypsin and the phagocytosis-positive cells were separated using a strong magnet, phagocytosis-negative cells were used as BAT-derived fibroblasts.

#### Pdgfrα-positive cell isolation

To isolate PDGFRα^+^ cells from WATi, magnetic-activated cell sorting (MACS) were used. For the best yield of PDGFRα^+^ cells WATi of three 8-week old WT C57Bl/6J mice was pooled. Tissue was minced and digested in digestion buffer (DMEM containing 0.5% BSA and 1.5 mg ml^−1^ Collagenase II). After digestion, all tissue debris was removed by filtration using a 100-µm nylon mesh (Merck Milipore, NY1H00010). Samples were centrifuged and the pellet was washed with 2 ml of ice-cold MACS buffer (0.5% BSA, 2 mM EDTA, 1% P/S in PBS pH 7.2). Cells were counted and FcR Blocking reagent and PDGFRα MicroBeads (Miltenyi Biotec, 130-101-502) were added to the cell suspension. After 15 min of incubation, cells were washed with MACS buffer, centrifuged and resuspended in MACS buffer. Liquid seperation columns (Miltenyi Biotec, 130-042-401) were rinsed with ice-cold MACS buffer and cell suspension was applied onto the column. The column was washed with MACS buffer and the flow through containing unlabelled cells was collected. PDGFRα^+^ cells were flushed out by pushing the plunger into the column after adding MACS buffer.

#### Cell culture of murine mesenchymal endothelial cells

Murine mesenchymal endothelial cells (Inscreenex, catalogue no. INS-CI-1004) were cultured following the manufacturer’s instructions (https://www.inscreenex.de/fileadmin/download/pdf/data-sheet/InstructionManual_muMEC.pdf).

#### Cell supernatants/conditioned medium

Cells were washed with Hank’s balanced salt solution (HBSS) (37 °C) (ThermoFisher, catalogue no. 14025-050), 300 µl of HBSS were added to each well and the cells were incubated in presence or absence of different stimuli (UV 200 mJ cm^−^^2^ (UVP, CL-1000 UV Crosslinker) for 10 min, 60 µM nutlin-3 (Cayman, 10004372) for 60 min, 1 µM l-(−)-NE (+)-bitartrate salt monohydrate (Sigma-Aldrich, A9512) for 60 min or 1 µM dipyridamole (Sigma-Aldrich, D9766) (Dip) for 60 min. Extracellular AMP, adenosine, inosine and hypoxanthine concentrations of the supernatants were measured with a ultra-high-performance liquid chromatogtaphy with tunable UV system (Waters) (CORTECS UPLC C18 Column (Waters), Empower v.3 software). ADP and ATP concentrations were measured using a ADP/ATP Ratio Assay Kit (Sigma-Aldrich, catalogue no. MAK135) and following the manufacturer’s instructions. Conditioned medium was applied on adipocytes for 16 h before RNA isolation.

#### Untargeted metabolomics of cell supernatant

Untargeted comparative metabolomics were executed using an ultra-performance liquid chromatographic system (Vanquish Flex, ThermoFisher Scientific) coupled to a high-resolution Orbitrap mass spectrometer (Orbitrap Exploris 120; ThermoFisher Scientific). Thermo Scientific Xcalibur v.4.4.16.2 software was used for data acquisition.

For sample generation, briefly, fresh supernatant was either immediately mixed with acetonitrile (1:5 (v/v)) for hydrophilic interaction separation or was inactivated by heat shock (67 °C, 10 min) for reversed phase separation, and all samples were further stored at −20 °C until analysis. Before LC–MS/MS analysis, the samples were filtrated (0.22 µm) by centrifugation and then stored in the autosampler at 4 °C. Extracted and filtrated samples were consecutively separated on four different analytical columns (Agilent HILICz, Waters Acquity C18, Phenomenex F5, ThermoFisher Scientific Accucore C30) each using electrospray positive and negative mode to cover a broad range of hydrophilic and lipophilic metabolites. Each sample set was accompanied by a matrix blank sample to remove background signals and a pooled sample as quality control to monitor intra-run variability. Analytes with less than 30% of standard deviation in the pooled samples were kept for further manual data curation and analysis. An internal mass calibration was performed in each run to ensure accurate mass results. The MS analysis alternated between MS1 mode (60.000 mass resolution) and data-dependent MS2 scans (30.000 mass resolution) using a scan range of 40–1,500 *m/z*. By means of a spectral library (*m/z* Cloud, ThermoFisher Scientific) containing >3,500 authentical compound spectra, metabolites were identified on the basis of the accurate mass to charge ratio (*m/z*), chromatographic data and MS/MS fragmentation patterns. Compound Discoverer v.3.2 software was used for untargeted analysis, peak identification and integration of mass spectrometric data. Qualitative pathway analysis, heatmap and volcano plot generation was done using MetaboAnalyst v.5.0 (https://www.metaboanalyst.ca). Metabolomics data were normalized to protein content.

#### Whole mount TUNEL staining

Tissue preparation and processing for whole mount staining was performed as described previously^42^ with some adaptations. In short, dissected BAT was fixed in 4% paraformaldehyde and cut to small pieces (2 × 2 × 2 mm). After several PBS washing steps, autofluorescence was quenched by incubation with 5% glycine (45 min). For blocking and permeabilization, the tissue pieces were incubated with 0.3% Triton X100 + 0.1% sodium citrate in 3% BSA in PBS for 2 h. The tissues were rinsed twice with PBS. To stain apoptotic nuclei, the tissue pieces were incubated in TUNEL reaction mixture for 2 h at 37 °C in the dark (In Situ Cell Death Detection Kit, 11684795910, Roche). After PBS washing, nuclei were stained with 4,6-diamidino-2-phenylindole (DAPI) for 15 min (5 µg ml^−1^). For staining of neutral lipids, tissue pieces were incubated with LipidTOX Deep Red Neutral Lipid stain (1:300 in PBS; H34477, Thermofisher) for 30 min. All steps were performed with continuous shaking. For microscopy, tissue pieces were transferred to a glass bottom dish (ibidi µ-Dish, ibidi GmbH) and imaged with a NikonA1 Ti confocal microscope (software NIS-Elements Advances Research, NIKON, RRID:SCR_014329).

#### Oil Red O staining

Differentiated adipocytes were washed twice with PBS, fixed with 4% paraformaldehyde at room temperature for 15 min and washed twice again with PBS. Then, cells were stained with 5 mg ml^−1^ oil red O in isopropanol (O0625, Sigma-Aldrich) at room temperature for 2 h. After that, cells were washed three times with tap water and left to dry at room temperature. For visualizing the scanner Epson Perfection V370 Photo was used.

#### Oxygen consumption rate measurements (Seahorse Mito Stress assay)

The oxygen consumption rate in adipocytes was measured to evaluate oxidative phosphorylation using the Agilent Seahorse XFe24 Analyzers (Agilent Technologies) following the manufacturer’s operating instructions. In brief, 2–4 × 10^4^ cells were seeded in wells of a 24-well XF Cell Culture Microplate (Agilent Technologies, 100777-004). Cells were grown in growth medium until they reached confluence and then they were differentiated into mature adipocytes. Assay was performed on mature adipocytes. The medium was exchanged to XF DMEM medium pH 7.4 (Agilent Technologies, 103575-100) with addition of following compounds: 25 mM glucose (G8270, Sigma-Aldrich), 2 mM glutamine (G9003, Sigma-Aldrich) and 2 mM sodium-pyruvate (P5280, Sigma-Aldrich). Cells were incubated in this medium for 1 h at 37 °C without CO_2_. Oxygen consumption rate measurements were performed with or without CL-316243 (10 µM) (C5976, Sigma-Aldrich) stimulation, which was followed by the sequential addition of 2 µM oligomycin (Complex V inhibitor), 1 µM FCCP and 0.5 µM rotenone/antimycin (Complex I/III inhibitor) (Agilent Technologies, 103015-100). All above experimental procedures were carried out at 37 °C. The basal and uncoupled respiration were determined and the results are expressed in pmol min^−1^ and normalized to the cell number.

#### Lipolysis assay

Differentiated adipocytes or adipose tissues explants were washed twice with lipolysis medium (Life Technologies, DMEM21603) supplemented with 2% w/v fatty acid–free BSA (Sigma-Aldrich, A7030) followed by incubation with lipolysis medium containing indicated substances (inosine (300 nM) (Sigma-Aldrich, I4125), l-(−)-NE (+)-bitartrate salt monohydrate (1 µM) (Sigma-Aldrich, A9512), 1 µM dipyridamole (Sigma-Aldrich, D9766) (Dip)) at 37 °C and 5% CO_2_ for two (murine adipocytes and tissues) or four (human adipocytes) hours. Cell culture media were collected, incubated 5 min at 37 °C with free glycerol reagent (Sigma-Aldrich, F6428) and absorption was measured at 540 nm. Glycerol release was calculated with glycerol standard (Sigma-Aldrich, G7793) and normalized to protein content or wet tissue weight.

#### Analysis of intracellular cAMP concentrations

Adipocytes were stimulated with or without adenosine 5′-monophosphate sodium salt (AMP) (300 nM) (Sigma-Aldrich, A1752), inosine (300 nM) (Sigma-Aldrich, I4125), hypoxanthine (300 nM) (Sigma-Aldrich, H9377) or l-(−)-NE (+)-bitartrate salt monohydrate (1 µM) (Sigma-Aldrich, A9512) for 15 min. Afterwards, cells were quickly washed with PBS and lysed with 0.1 M HCl. Subsequently, samples were analysed using Direct cAMP ELISA Kit (Enzo, ADI-901-066), following the manufacturer’s instructions. Measurement of optical density was performed at 405 nm using a plate reader (Perkin Elmer).

#### Western blots

Proteins were isolated using lysis buffer (50 mM Tris, pH 7.5, 150 mM sodium chloride, 1% NP-40, 0.5% sodium deoxycholate, 0.1% SDS, 0.1 mM EDTA and 0.1 mM EGTA) supplemented with complete protease inhibitor cocktail (Roche), 1 mM Na_3_VO_4_ and 10 mM NaF. Protein amount from all samples was quantified using Bradford assay followed by concentration normalization before western blot experiments. Western blot was carried out following standard procedures (molecular weight markers: Colour Prestained Protein Standard, New England BioLabs, P7712S, P7719S). As primary Antibodies UCP1 (Cell Signaling, 14670S, 1:1,000; custom made, 1:1,000), ENT1 (Antibodies online, ABIN387941, 1:500) and Calnexin (EMD Millipore Corp, 208880, 1:1,000), Phospho-p38 MAPK (T180/Y182) (Cell Signaling; order no. 9211S, 1:1,000), Phospho Creb (Ser133) (Cell Signaling; order no. 9198S, 1:1,000), Phospho ATF2 (Thr71) (Cell Signaling; order no. 9221S, 1:1,000) were applied. Proteins were visualized using an ImageQuant LAS 4000 chemiluminescence reader and enhanced chemiluminescence reagent or an Odyssey Fc Imaging System (LI-COR Bioscience) with fluorescence-labelled secondary antibodies (antirabbit IgG (H+L): Dylight 800, 4× PEG Conjugate, Cell Signaling Technology, 1:10,000), according to the manufacturer’s protocol. Bands were analysed and quantified with Image Studio Lite v.5.2 software. For uncropped source blots, please see Supplementary information. Expression values were normalized to Calnexin expression.

#### RNA isolation and quantitative PCR

Total RNA was isolated using innuSOLV RNA reagent (Analytik Jena, 845-SB-2090100) and reverse transcribed with the complementary DNA synthesis kit (NEB, ProtoScript II First Strand cDNA Synthesis Kit). Quantitative PCR with reverse transcription was performed with SYBR Green Master Mix (ThermoFisher Scientific, 4309155) using an Applied Biosystems machine (ThermoFisher Scientific). Expression levels were calculated as delta Ct values and normalized to the housekeeping gene *Hprt/HPRT*. For the lists of murine and humane primer sequences used for real-time PCR, please see Supplementary Tables 1 and 2. Primer pair quality was assessed by analysing the melting curves.

To study the effect of inosine treatment on adipocytes’ mRNA expression, cells were incubated for 16 h with 300 nM inosine (Sigma-Aldrich, I4125), before RNA isolation. To study the effect of secreted factors on adipocytes’ mRNA expression, cells were incubated for 16 h with respective supernatants, before RNA isolation.

#### RNA-sequencing

RNA-sequencing (RNA-seq) was performed using standard next-generation sequencing bulk 3′ poly(A)-mRNA sequencing.

#### Gene expression analysis of apoptotic genes

To isolate RNA from cell fractions or whole tissue, the NucleoSpin RNA II kit (Macherey & Nagel, 740933) was used. Quantitative real-time PCR was performed after cDNA synthesis as described^43^. Relative gene expression was normalized to housekeeper 36b4 mRNA using the 2^-ΔΔCt^ method. For the list of the TaqMan assays used, please see Supplementary Table 3.

#### Phosphoproteomics analysis

Cells were treated with 300 nM inosine (Sigma-Aldrich, order no. I4125) or 1 µM FORSK (Sigma, order no. F6886) for 15 min. Afterwards, cells were washed twice with cold tris-buffered saline and collected in 500 µl of 4% SDC buffer (4% SDC (Sigma, order no. 30970), and 100 mM Tris (Sigma, order no. AE15.3) pH 8.5) and boiled for 5 min at 95 °C.

Phosphopeptides were enriched using the Easyphos workflow^44^ with 1 mg of protein input. For MS analysis peptides were loaded onto a 50-cm column at 60 °C with a 75 µM inner diameter, packed in-house with 1.9 µM C18 ReproSil particles (Dr. Maisch GmbH). Peptides were separated on a 120 min gradient by reversed phase chromatography using a binary buffer system consisting of 0.1% formic acid (buffer A) and 80% ACN in 0.1% formic acid (buffer B). Mass spectra were acquired on a Thermo Orbitrap Exploris 480 mass spectrometer. Acquisition was performed using a data-dependent 1 s cycle time method with a maximum injection time of 80 ms, a scan range of 300–1,650 Th, and an AGC target of 300% without field asymmetric ion mobility spectrometry. Sequencing was performed via higher energy collisional dissociation fragmentation with a target value of 1 × 10^5^, and a window of 1.4 Th. Survey scans were acquired at a resolution of 60,000. Resolution for high-collision dissociation spectra was set to 15,000 with a maximum ion injection time of 50 ms. Dynamic exclusion was set to 40 s, and apex trigger was enabled. Raw mass spectrometry data were processed with MaxQuant v.2.0.1.0 with ‘match between runs’ enabled. ‘Max. missed cleavages’ were set to 2. Default settings were used if not stated otherwise. Statistical analysis and imputation of missing values was performed with Perseus software v.1.6.13.0. Multiplicity (the number of phosphorylations at the detected peptide) is given as *M*. Phosphoproteomics data are available at PRIDE PXD032153.

#### ^3^H-inosine uptake of adipocytes

Cells were washed and incubated at 37 °C with HBSS (ThermoFisher, 14025-050). Subsequently, 1 µCi 3H-inosine (Hartmann Analytic, ART0738) per well was added and the cells were incubated for 5 min. ^3^H of the supernatant was counted using a Beckman Counter. The cells were washed with PBS, lysed with Triton X dilution (VWR, 28817.295; 1:1000 in HBSS) and ^3^H of the lysate was counted. Data were normalized to the protein concentrations of respective wells.

#### Knockout of SLC29A1 in human adipocytes (CRISPR–Cas9)

SLC29A1 was knocked out in human adipocytes using CRISPR–Cas9 system. The CRISPR guide RNAs (gRNAs) were purchased from GenScript (SC1805, Species: human: 1. SLC29A1 CRISPR guide RNA 1, gRNA target sequence: GCAGGATCCCCCAGTCCGTA; 2. SLC29A1 CRISPR gRNA 2, gRNA target sequence: CAGGCTGCCCAGGATCCGTA; 3. SLC29A1 CRISPR guide RNA 3, gRNA target sequence: GCAGTATTTCACAAACCGCC) in a pLentiCRISPR v2 plasmid format. The SLC29A1 CRISPR guide RNA 3 was chosen for the experiments with human adipocytes. Lentiviral particles were produced by the lentiviral vector platform of the Institute of Pharmacology and Toxicology, University of Bonn, following standard procedures^45^. A non-targeting construct (gRNA: GCATAACGGCCGAGCACCAC) was used for production of control virus. Human adipocytes were transduced with lentiviral particles 8 h after seeding and afterwards the cells were differentiated following the protocols described above.

#### Overexpression of SLC29A1 variants in human embryonic kidney 293T (HEK293T) cells

Plasmids with inserts of either the WT or rs45573936 variant of SLC29A1 were ordered from GeneScript (target vector name pcDNA3.1(−)). HEK293T cells (ATCC, CRL-3216) were transfected (standard calcium phosphate transfection method) with respective plasmids 16 h after seeding. Experiments were performed 2 days after transfection.

### Analysis of the *SLC29A1* Ile216Thr (rs45573936) variant in human participants

#### Description

The cohort analysed for BMI association of Ile216Thr (rs45573936) variants derived from a self-contained population of Sorbs in Germany described previously^29^.

The study has been approved by the Ethics Committee of the University of Leipzig (reg. no. 088-2005) and is in accordance with the Declaration of Helsinki. All participants gave written informed consent before taking part in the study.

#### Genotyping of the Ile216Thr (rs45573936) variant

Genotyping of the Ile216Thr (rs45573936) variant was performed using the LightCycler480 system (Roche Diagnostics) according to the manufacturer’s protocol. First, PCR was run with initial denaturation at 95 °C for 6 min, followed by 45 cycles of 20 s denaturation at 95 °C, 40 s annealing at 62 °C and 90 s of primer extension at 72 °C followed by final extension for 7 min at 72 °C. PCR was conducted using the Taq PCR Core Kit 1000 units (Qiagen) and 0.1 mM forward and 0.1 mM reverse primers in a total volume of 20 µl. For the rs4553936 variant asymmetric PCRs were performed with 0.2 mM reverse primer. Primers and probes were synthesized by TIB Molbiol. The generated PCR products were taken for genotyping with 50 nM (final) of probe oligomers by melting curve analysis with the following protocol: 95 °C for 60 s, 40 °C for 60 s, continuous increase to 70 °C with a ramp rate of 0.19 °C s^−1^. Call rate for the variant was 99%. For quality control, 1.8% of all samples were genotyped in duplicates blinded to the investigator. Resulting concordance rate was 99%.

### Analysis of human visceral and subcutaneous WATs

#### Human data

The human cohort comprises adipose tissues from 2,044 individuals of the Leipzig Obesity Biobank. Omental visceral adipose tissue samples were collected from 1,581 individuals classified as normal weight (*n* = 58, mean age 60.5 ± 14.8 years, mean BMI 22.5 ± 1.9 kg m^−2^), overweight (*n* = 56, mean age 65.0 ± 12.7 years, mean BMI 27.2 ± 1.4 kg m^−2^) or obesity (*n* = 1,467, mean age 47.1 ± 11.7 years, mean BMI 48.8 ± 8.4 kg m^−2^). Abdominal subcutaneous adipose tissue samples with normal weight (*n* = 47, mean age 64.5 ± 13 years, mean BMI 22.9 ± 1.7 kg m^−2^), overweight (*n* = 56, mean age 62.7 ± 13.5 years, mean BMI 27.3 ± 1.5 kg m^−2^) or obesity (*n* = 1,372, mean age 47.1 ± 12 years, mean BMI 48.8 ± 8.6 kg m^−2^) were obtained from 1,475 individuals. Of these, paired subcutaneous and visceral data are from 1,013 patients. Adipose tissue samples were collected during elective laparoscopic abdominal surgery as described^46^, immediately frozen in liquid nitrogen and stored at −80 °C. The study was performed in agreement with the Declaration of Helsinki and approved by the Ethics Committee of the University of Leipzig (approval number 159-12-21052012). All participants gave written informed consent before taking part in the study. Body composition and metabolic parameters were measured as previously described^47^.

#### RNA-seq

Single-end and ribosomal RNA-depleted RNA-seq data were prepared on the basis of the SMARTseq protocol^48,49^. In brief, RNA was enriched and reverse transcribed by Oligo(dT) and TSO primers. In silico PCR primers were used for cDNA amplification and cDNA were processed with Tn5 using Nextera DNA Flex kit. All libraries were sequenced on a Novaseq 6000 instrument at Functional Genomics Center Zurich. Adaptor and quality trimming of the raw reads were computed using Fastp (v.0.20.0, ref. ^50^) considering a minimum read length of 18 nts and a quality cut-off of 20. Reads were mapped against the human genome (GRCh38.p13) using the STAR algorithm (v.2.7.4a, ref. ^51^), permitting 50 multiple alignments per read. FeatureCounts (v.2.0.1, ref. ^52^) was applied to assign genomic features to mapped reads, counting multiple mapped reads fractionally. Count data were homoscedastic normalized with respect to library size using the variance stabilizing transformation from DESeq2 (v.1.32.0, ref. ^53^). As read depth and sex of the data represented the largest source of variance, an adjustment was performed to account for this batches.

### Quantification and statistical analysis

To determine the group size necessary for sufficient statistical power, power analysis was carried out with PS Power and Sample Size Calculation Software using preliminary data and all experiments were designed and powered to a minimum of 0.8 as calculated.

Mice were allocated randomly into experimental groups. Owing to the nature of the cell culture experiments, randomization of the samples was not applicable. As most studies were performed by individual researchers knowing the design of the studies, blinding during data collection and analysis was not performed.

Two-tailed *t*-tests were used for single comparisons and analysis of variance (ANOVA) with Tukey’s post hoc tests for multiple comparisons. *P* values below 0.05 were considered significant. Statistical analysis and data plotting was performed with GraphPad Prism v.6 software. Unless otherwise specified, *n* defines the number of animals or cell cultures analysed. Data are represented as single data points or dot plots with mean ± s.e.m. Please refer to figure legends for description of sample sizes and statistical tests performed.

Statistical evaluation of the metabolomics data was executed using Compound Discoverer (v.3.2, ThermoFisher). Hypothesis test was performed by a one-way ANOVA model with Tukey’s post hoc test. *P* values are adjusted by Benjamini–Hochberg algorithm.

Correlations between gene expression of visceral and subcutaneous WATs of the human cohort were calculated using the R packages ggpubr (v.0.4.0)^54^, based on the Spearman correlation coefficient, and a confidence interval of 0.95. *P* values were corrected for multiple inference using the Holm method. Analyses were performed under R v.4.1.

### Reporting summary

Further information on research design is available in the Nature Research Reporting Summary linked to this paper.

## Online content

Any methods, additional references, Nature Research reporting summaries, source data, extended data, supplementary information, acknowledgements, peer review information; details of author contributions and competing interests; and statements of data and code availability are available at 10.1038/s41586-022-05041-0.

## Supplementary information

Supplementary Information This file contains the uncropped blots. **a**–**c**, Uncropped immunoblots of Extended Data Fig. 2e: P-p38 (left) and Calnexin (right), respectively. **d**–**f**, Uncropped immunoblots of Extended Data Fig. 2f: P-ATF2 (left) and Calnexin (right), respectively. **g**–**i**, Uncropped immunoblots of Extended Data Fig. 2g: P-Creb (left) and Calnexin (right), respectively. **j**,**k**, Uncropped immunoblots of Extended Data Fig. 3d. **j**, Calnexin (top band) and UCP1 (bottom band). **k**, UCP1 (left) and Calnexin (right). **l-n**, Uncropped immunoblots of Extended Data Fig. 6d and merged pictures of the immunoblots and respective molecular weight markers: ENT1 (left) and Calnexin (right), respectively. All samples framed in red are represented in the original figure and were quantified. All samples framed in blue were quantified.

Reporting Summary

Supplementary Fig. 1 Qualitative enrichment analysis of metabolic pathways. Untargeted metabolomics of murine brown adipocytes after nutlin-3 treatment: qualitative enrichment analysis of metabolic pathways of secreted metabolites based on the Kyoto Encyclopedia of Genes and Genomes metabolic pathways (*n* = 6). One-way ANOVA with Tukey’s post hoc test.

Supplementary Tables This file contains Supplementary Tables 1–3, which list murine primer sequences, human primer sequences and TaqMan assays.

## Data Availability

Source data are provided with this paper. Exact *P* values are also included within the Source Data file. Further details on datasets and protocols that support the findings of this study will be made available by the corresponding author upon reasonable request (email Alexander.Pfeifer@uni-bonn.de). Phosphoproteomic data are available at PRIDE PXD032153. GRCh38.p13 database (publicly available) was used to map the RNA-seq data of human adipose tissues against the human genome.

## Source data

Source Data Figs. 1–3 and Extended Data Figs. 1–7.

## Extended data

are available for this paper at 10.1038/s41586-022-05041-0.

## References

[1] Kajimura S, Spiegelman BM, Seale P (2015). Brown and beige fat: physiological roles beyond heat generation. Cell Metab..

[2] Scheele C, Wolfrum C (2020). Brown adipose crosstalk in tissue plasticity and human metabolism. Endocr. Rev..

[3] Becher T (2021). Brown adipose tissue is associated with cardiometabolic health. Nat. Med..

[4] Pellegrinelli V, Carobbio S, Vidal-Puig A (2016). Adipose tissue plasticity: how fat depots respond differently to pathophysiological cues. Diabetologia.

[5] Graja A, Gohlke S, Schulz TJ (2019). Aging of brown and beige/brite adipose tissue. Handb. Exp. Pharmacol..

[6] Himms-Hagen J (1989). Brown adipose tissue thermogenesis and obesity. Prog. Lipid Res..

[7] Nedergaard J, Wang Y, Cannon B (2019). Cell proliferation and apoptosis inhibition: essential processes for recruitment of the full thermogenic capacity of brown adipose tissue. Biochim. Biophys. Acta, Mol. Cell. Biol. Lipids.

[8] Schlein C (2021). Endogenous fatty acid synthesis drives brown adipose tissue involution. Cell Rep..

[9] Cypess AM (2009). Identification and importance of brown adipose tissue in adult humans. N. Engl. J. Med..

[10] van Marken Lichtenbelt WD (2009). Cold-activated brown adipose tissue in healthy men. N. Engl. J. Med..

[11] Elliott MR (2009). Nucleotides released by apoptotic cells act as a find-me signal to promote phagocytic clearance. Nature.

[12] Hotamisligil GS, Davis RJ (2016). Cell signaling and stress responses. Cold Spring Harb. Perspect. Biol..

[13] Roh HC (2018). Warming induces significant reprogramming of beige, but not brown, adipocyte cellular identity. Cell Metab..

[14] Medina CB (2020). Metabolites released from apoptotic cells act as tissue messengers. Nature.

[15] Vassilev LT (2004). In vivo activation of the p53 pathway by small-molecule antagonists of MDM2. Science.

[16] Cannon B, Nedergaard J (2004). Brown adipose tissue: function and physiological significance. Physiol. Rev..

[17] Xu Z, Liu J, You W, Wang Y, Shan T (2019). Cold exposure induces nuclear translocation of CRTC3 in brown adipose tissue. J. Cell. Biochem..

[18] Labbe SM (2016). mTORC1 is required for brown adipose tissue recruitment and metabolic adaptation to cold. Sci. Rep..

[19] Robidoux J (2006). Maximal beta3-adrenergic regulation of lipolysis involves Src and epidermal growth factor receptor-dependent ERK1/2 activation. J. Biol. Chem..

[20] Cao W (2004). p38 mitogen-activated protein kinase is the central regulator of cyclic AMP-dependent transcription of the brown fat uncoupling protein 1 gene. Mol. Cell. Biol..

[21] Paulo E (2018). Sympathetic inputs regulate adaptive thermogenesis in brown adipose tissue through cAMP-Salt inducible kinase axis. Sci. Rep..

[22] Fredholm BB, AP IJ, Jacobson KA, Linden J, Muller CE (2011). International Union of Basic and Clinical Pharmacology. LXXXI. Nomenclature and classification of adenosine receptors–an update. Pharmacol. Rev..

[23] Harms M, Seale P (2013). Brown and beige fat: development, function and therapeutic potential. Nat. Med..

[24] Ward JL, Sherali A, Mo ZP, Tse CM (2000). Kinetic and pharmacological properties of cloned human equilibrative nucleoside transporters, ENT1 and ENT2, stably expressed in nucleoside transporter-deficient PK15 cells. Ent2 exhibits a low affinity for guanosine and cytidine but a high affinity for inosine. J. Biol. Chem..

[25] Seki T (2016). Endothelial PDGF-CC regulates angiogenesis-dependent thermogenesis in beige fat. Nat. Commun..

[26] Grenz A (2012). Equilibrative nucleoside transporter 1 (ENT1) regulates postischemic blood flow during acute kidney injury in mice.. J. Clin. Invest..

[27] Gnad T (2014). Adenosine activates brown adipose tissue and recruits beige adipocytes via A2A receptors. Nature.

[28] Jespersen NZ (2013). A classical brown adipose tissue mRNA signature partly overlaps with brite in the supraclavicular region of adult humans. Cell Metab..

[29] Tonjes A (2010). Association of FTO variants with BMI and fat mass in the self-contained population of Sorbs in Germany. Eur. J. Hum. Genet..

[30] Schulz TJ, Tseng YH (2013). Systemic control of brown fat thermogenesis: integration of peripheral and central signals. Ann. NY Acad. Sci..

[31] Villarroya F, Cereijo R, Villarroya J, Giralt M (2017). Brown adipose tissue as a secretory organ. Nat. Rev. Endocrinol..

[32] Ruan CC (2018). A2A receptor activation attenuates hypertensive cardiac remodeling via promoting brown adipose tissue-derived FGF21. Cell Metab..

[33] Welihinda AA, Kaur M, Greene K, Zhai Y, Amento EP (2016). The adenosine metabolite inosine is a functional agonist of the adenosine A2A receptor with a unique signaling bias. Cell Signal.

[34] Cypess AM (2015). Activation of human brown adipose tissue by a beta3-adrenergic receptor agonist. Cell Metab..

[35] Choi DS (2004). The type 1 equilibrative nucleoside transporter regulates ethanol intoxication and preference. Nat. Neurosci..

[36] Chen JF (1999). A(2A) adenosine receptor deficiency attenuates brain injury induced by transient focal ischemia in mice. J. Neurosci..

[37] Eckle T (2007). Cardioprotection by ecto-5'-nucleotidase (CD73) and A2B adenosine receptors. Circulation.

[38] Gnad T (2020). Adenosine/A2B receptor signaling ameliorates the effects of aging and counteracts obesity. Cell Metab.

[39] Haas B (2009). Protein kinase G controls brown fat cell differentiation and mitochondrial biogenesis. Sci Signal.

[40] Rodriguez AM, Elabd C, Amri EZ, Ailhaud G, Dani C (2005). The human adipose tissue is a source of multipotent stem cells. Biochimie.

[41] Chen Y (2013). miR-155 regulates differentiation of brown and beige adipocytes via a bistable circuit. Nat. Commun..

[42] Fischer K (2017). Alternatively activated macrophages do not synthesize catecholamines or contribute to adipose tissue adaptive thermogenesis. Nat. Med..

[43] Fischer AW (2021). Lysosomal lipoprotein processing in endothelial cells stimulates adipose tissue thermogenic adaptation. Cell Metab..

[44] Humphrey SJ, Karayel O, James DE, Mann M (2018). High-throughput and high-sensitivity phosphoproteomics with the EasyPhos platform. Nat. Protoc..

[45] Pfeifer A, Brandon EP, Kootstra N, Gage FH, Verma IM (2001). Delivery of the Cre recombinase by a self-deleting lentiviral vector: efficient gene targeting in vivo. Proc. Natl Acad. Sci. USA.

[46] Langhardt J (2018). Effects of weight loss on glutathione peroxidase 3 serum concentrations and adipose tissue expression in human obesity. Obes. Facts.

[47] Kloting N (2010). Insulin-sensitive obesity. Am. J. Physiol. Endocrinol. Metab..

[48] Picelli S (2014). Full-length RNA-seq from single cells using Smart-seq2. Nat. Protoc..

[49] Song Y (2018). A comparative analysis of library prep approaches for sequencing low input translatome samples. BMC Genomics.

[50] Chen S, Zhou Y, Chen Y, Gu J (2018). fastp: an ultra-fast all-in-one FASTQ preprocessor. Bioinformatics.

[51] Dobin A (2013). STAR: ultrafast universal RNA-seq aligner. Bioinformatics.

[52] Wingett SW, Andrews S (2018). FastQ Screen: a tool for multi-genome mapping and quality control. F1000Res.

[53] Love MI, Huber W, Anders S (2014). Moderated estimation of fold change and dispersion for RNA-seq data with DESeq2. Genome Biol.

[54] Kassambara, A. & Kassambara, M. A. Package ‘ggpubr’. R package version 0.1 6 (R Foundation for Statistical Computing, 2020).

