# Supplementary material for: Apoptotic brown adipocytes enhance energy expenditure via extracellular inosine
Source: Nature. 2022 Jul 5;609(7926):361–8. doi: 10.1038/s41586-022-05041-0 (PMC9452294; doi:10.1038/s41586-022-05041-0)
Supplement: Supplementary file 1 — This file contains the uncropped blots. a–c, Uncropped immunoblots of Extended Data Fig. 2e: P-p38 (left) and Calnexin (right), respectively. d–f, Uncropped immunoblots of Extended Data Fig. 2f: P-ATF2 (left) and Calnexin (right), respectively. g–i, Uncropped immunoblots of Extended Data Fig. 2g: P-Creb (left) and Calnexin (right), respectively. j,k, Uncropped immunoblots of Extended Data Fig. 3d. j, Calnexin (top band) and UCP1 (bottom band). k, UCP1 (left) and Calnexin (right). l-n, Uncropped immunoblots of Extended Data Fig. 6d and merged pictures of the immunoblots and respective molecular weight markers: ENT1 (left) and Calnexin (right), respectively. All samples framed in red are represented in the original figure and were quantified. All samples framed in blue were quantified. [file 41586_2022_5041_MOESM1_ESM.pdf]

nature portfolio

https://doi.org/10.1038/s41586-022-05041-0

Supplementary information

Apoptotic brown adipocytes enhance energy expenditure via extracellular inosine

In the format provided by the authors and unedited

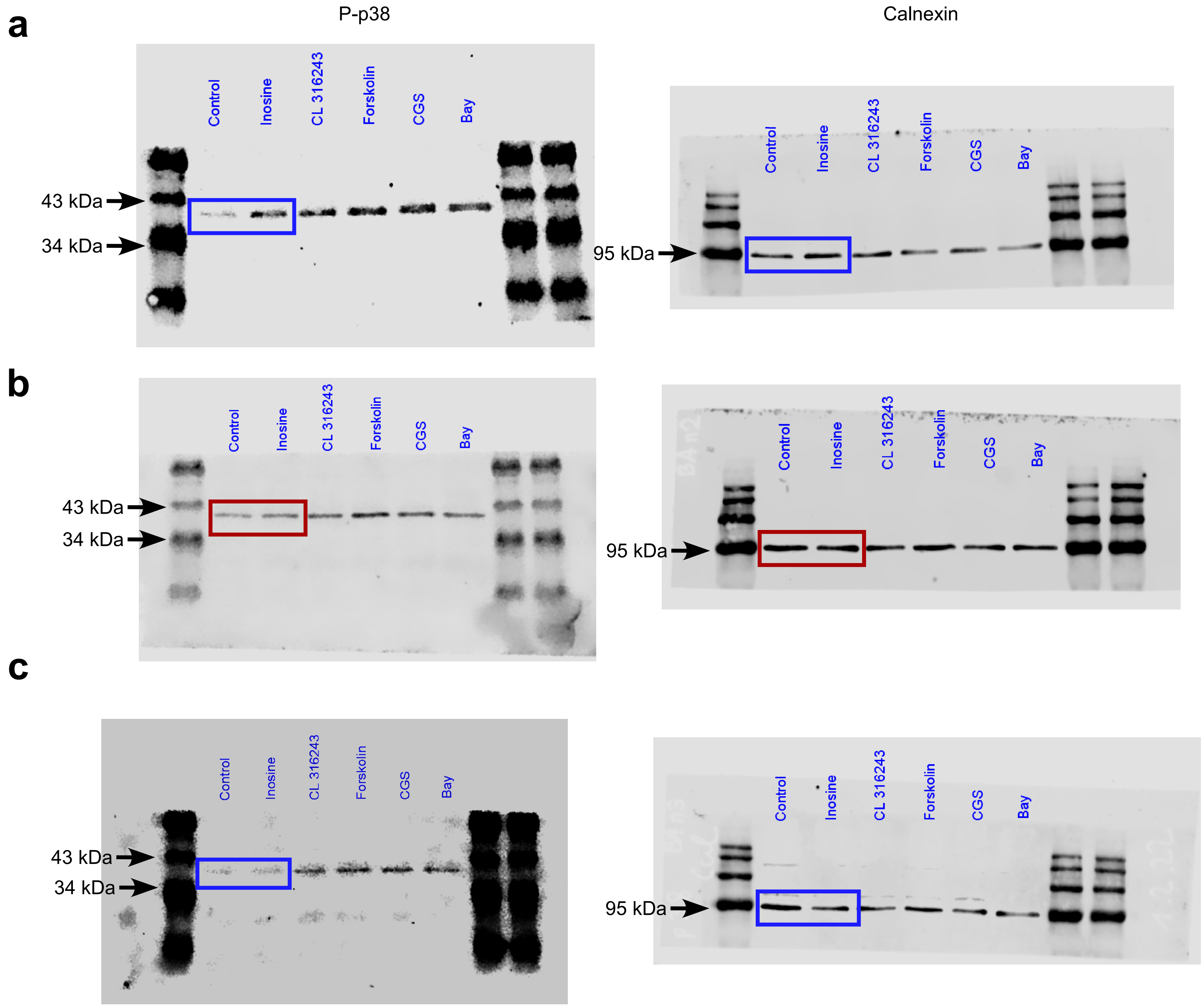
a
P-p38
Calnexin
Control
Inosine
CL 316243
Forskolin
CGS
Bay
43 kDa
34 kDa
95 kDa
b
Control
Inosine
CL 316243
Forskolin
CGS
Bay
43 kDa
34 kDa
95 kDa
c
Control
Inosine
CL 316243
Forskolin
CGS
Bay
43 kDa
34 kDa
95 kDa

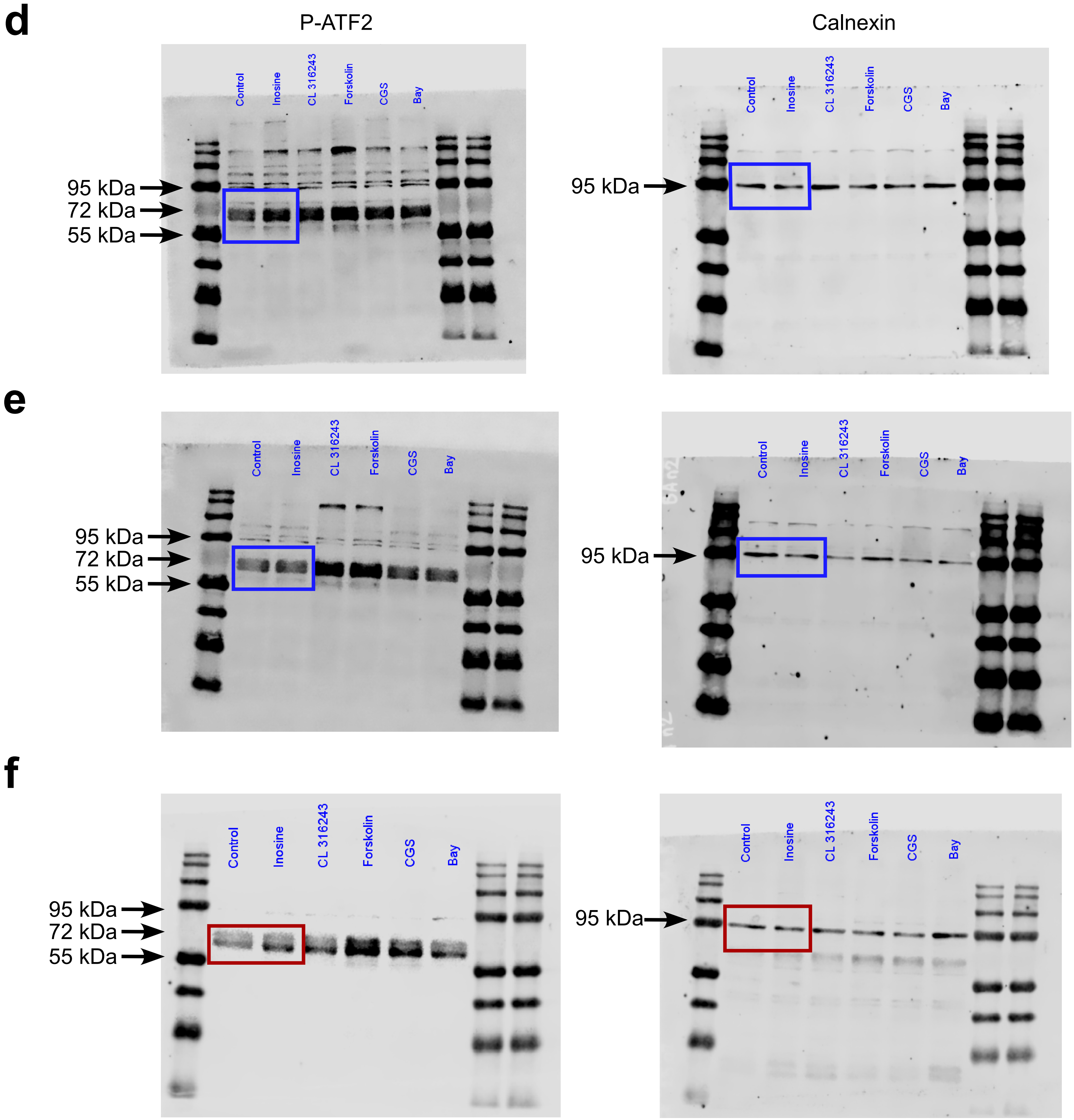
d
P-ATF2
Calnexin
Control
Inosine
CL 316243
Forskolin
CGS
Bay
95 kDa
72 kDa
55 kDa
e
f

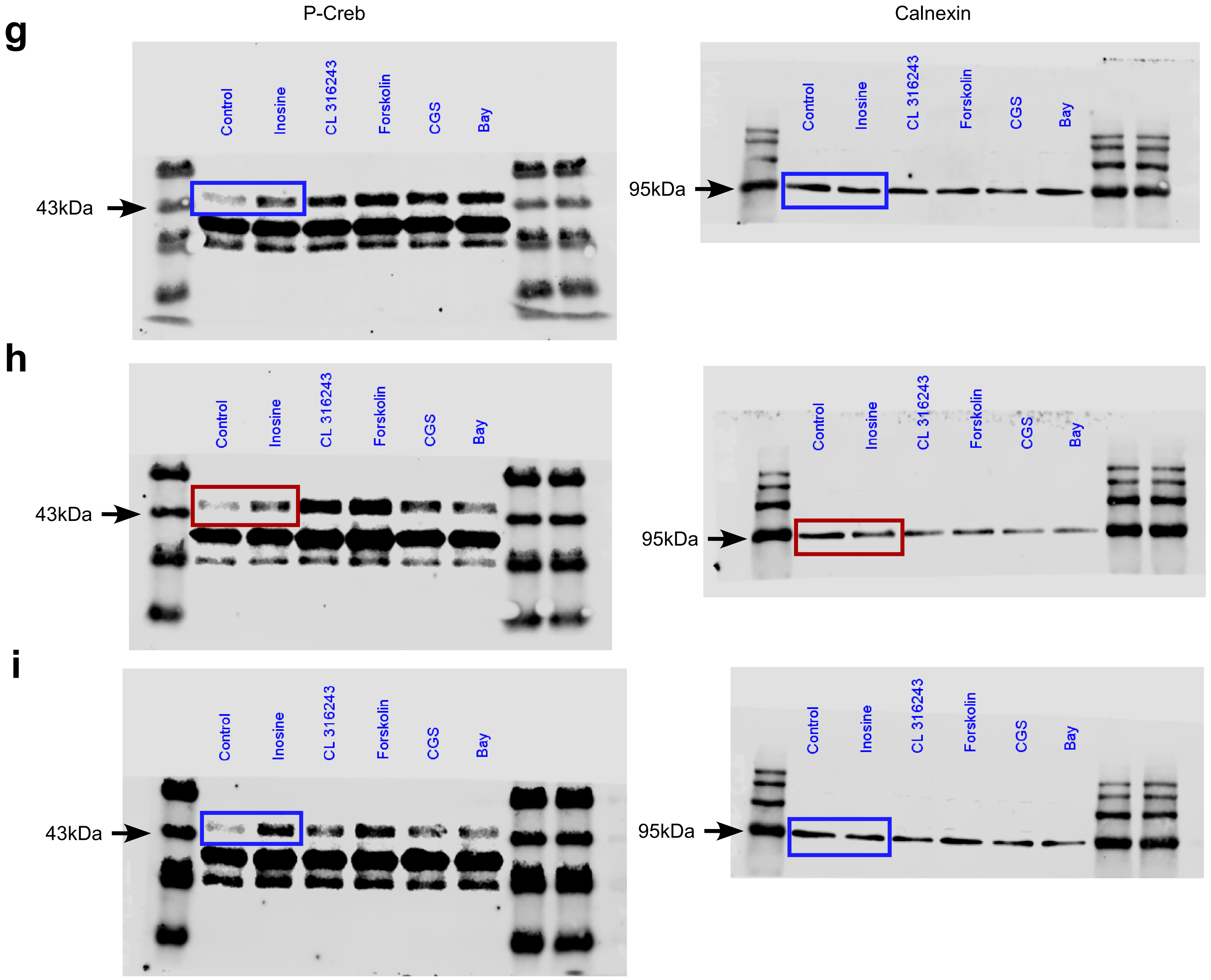
g
P-Creb
Calnexin
Control
Inosine
CL 316243
Forskolin
CGS
Bay
43kDa
95kDa
h
43kDa
95kDa
i
43kDa
95kDa

j

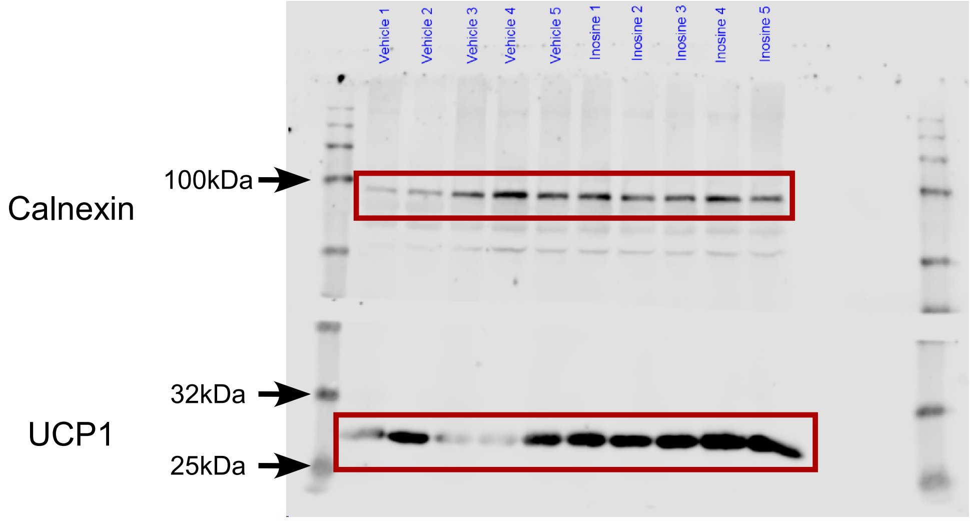
Calnexin
100kDa
UCP1
32kDa
25kDa
Vehicle 1
Vehicle 2
Vehicle 3
Vehicle 4
Vehicle 5
Inosine 1
Inosine 2
Inosine 3
Inosine 4
Inosine 5

k

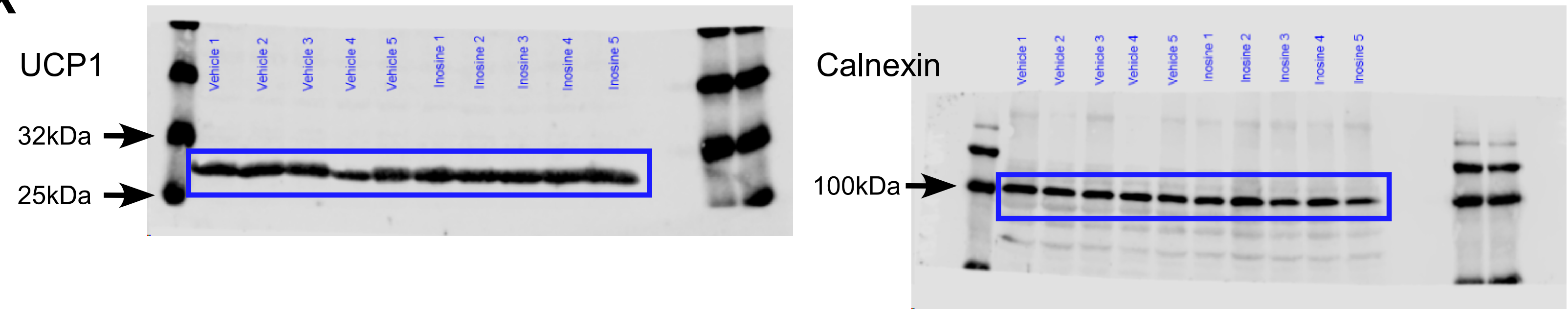
UCP1
32kDa
25kDa
Vehicle 1
Vehicle 2
Vehicle 3
Vehicle 4
Vehicle 5
Inosine 1
Inosine 2
Inosine 3
Inosine 4
Inosine 5
Calnexin
100kDa
Vehicle 1
Vehicle 2
Vehicle 3
Vehicle 4
Vehicle 5
Inosine 1
Inosine 2
Inosine 3
Inosine 4
Inosine 5

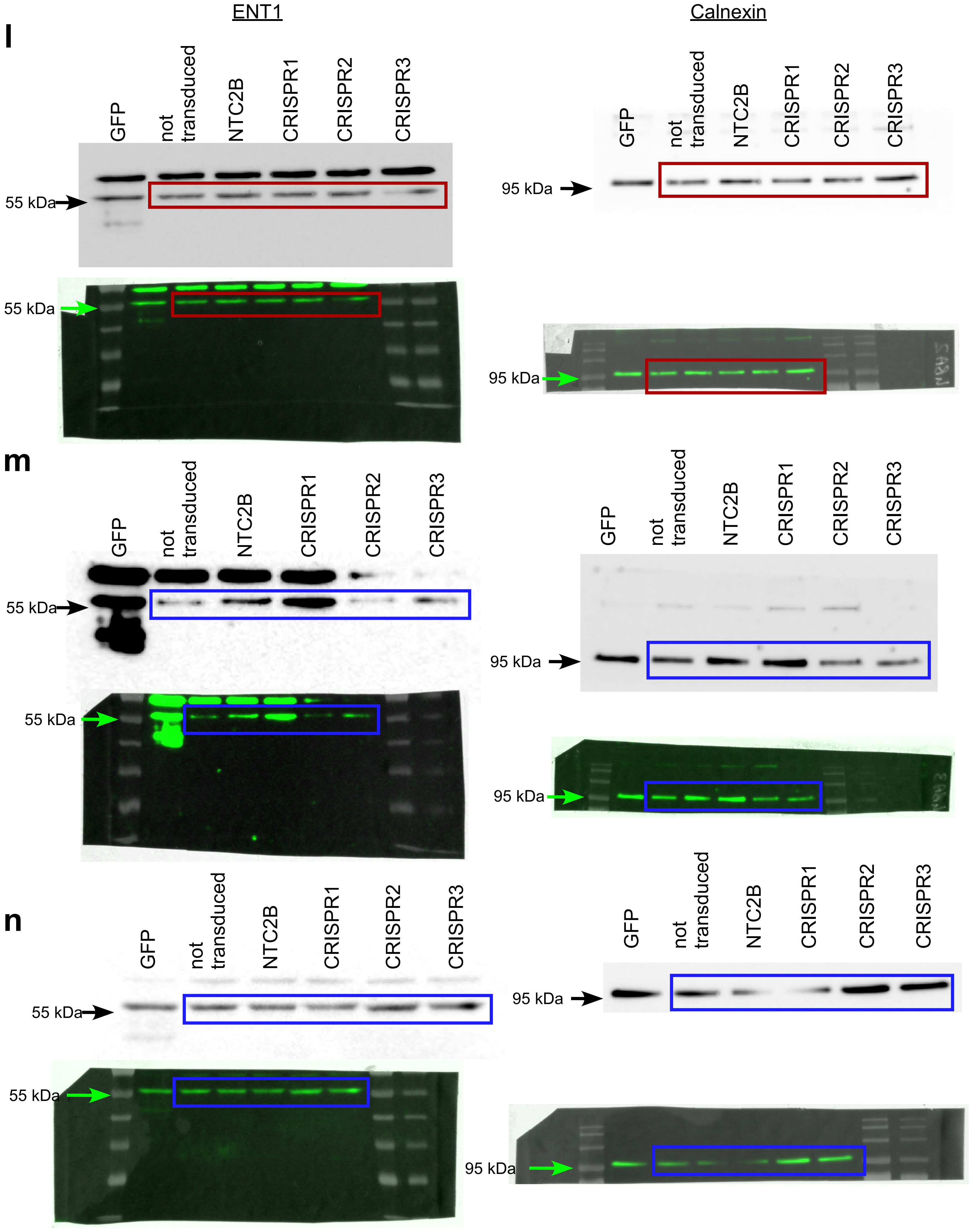
ENT1
Calnexin
l
GFP
not transduced
NTC2B
CRISPR1
CRISPR2
CRISPR3
55 kDa
55 kDa
GFP
not transduced
NTC2B
CRISPR1
CRISPR2
CRISPR3
95 kDa
95 kDa
m
GFP
not transduced
NTC2B
CRISPR1
CRISPR2
CRISPR3
55 kDa
55 kDa
GFP
not transduced
NTC2B
CRISPR1
CRISPR2
CRISPR3
95 kDa
95 kDa
n
GFP
not transduced
NTC2B
CRISPR1
CRISPR2
CRISPR3
55 kDa
55 kDa
GFP
not transduced
NTC2B
CRISPR1
CRISPR2
CRISPR3
95 kDa
95 kDa

Supplementary Information

a-c Uncropped immunoblots of Extended Data Fig. 2e: P-p38 (left) and Calnexin (right), respectively. **d-f** Uncropped immunoblots of Extended Data Fig. 2f: P-ATF2 (left) and Calnexin (right), respectively. **g-i** Uncropped immunoblots of Extended Data Fig. 2g: P-Creb (left) and Calnexin (right), respectively. **j, k** Uncropped immunoblots of Extended Data Fig. 3d: **j** Calnexin (top band) and UCP1 (bottom band), **k** UCP1 (left) and Calnexin (right). **l-n** Uncropped immunoblots of Extended Data Fig. 6d and merged pictures of the immunoblots and respective molecular weight markers: ENT1 (left) and Calnexin (right), respectively.

All samples framed in red are represented in the original figure and were quantified. All samples framed in blue were quantified.
