# Supplementary material for: Apoptotic brown adipocytes enhance energy expenditure via extracellular inosine
Source: Nature. 2022 Jul 5;609(7926):361–8. doi: 10.1038/s41586-022-05041-0 (PMC9452294; doi:10.1038/s41586-022-05041-0)
Supplement: Supplementary file 2 — Reporting Summary [file 41586_2022_5041_MOESM2_ESM.pdf]

nature portfolio

Corresponding author(s): Alexander Pfeifer

Last updated by author(s): 2022/07/26

Reporting Summary

Nature Portfolio wishes to improve the reproducibility of the work that we publish. This form provides structure for consistency and transparency in reporting. For further information on Nature Portfolio policies, see our Editorial Policies and the Editorial Policy Checklist.

Please do not complete any field with "not applicable" or n/a. Refer to the help text for what text to use if an item is not relevant to your study.
For final submission: please carefully check your responses for accuracy; you will not be able to make changes later.

Statistics

For all statistical analyses, confirm that the following items are present in the figure legend, table legend, main text, or Methods section.

n/a	Confirmed	
☐	☒	The exact sample size (n) for each experimental group/condition, given as a discrete number and unit of measurement
☒	☐	A statement on whether measurements were taken from distinct samples or whether the same sample was measured repeatedly
☐	☒	The statistical test(s) used AND whether they are one- or two-sided<br>*Only common tests should be described solely by name; describe more complex techniques in the Methods section.*
☐	☒	A description of all covariates tested
☐	☒	A description of any assumptions or corrections, such as tests of normality and adjustment for multiple comparisons
☐	☒	A full description of the statistical parameters including central tendency (e.g. means) or other basic estimates (e.g. regression coefficient) AND variation (e.g. standard deviation) or associated estimates of uncertainty (e.g. confidence intervals)
☐	☒	For null hypothesis testing, the test statistic (e.g. F, t, r) with confidence intervals, effect sizes, degrees of freedom and P value noted<br>*Give P values as exact values whenever suitable.*
☒	☐	For Bayesian analysis, information on the choice of priors and Markov chain Monte Carlo settings
☒	☐	For hierarchical and complex designs, identification of the appropriate level for tests and full reporting of outcomes
☐	☒	Estimates of effect sizes (e.g. Cohen's d, Pearson's r), indicating how they were calculated

Our web collection on statistics for biologists contains articles on many of the points above.

Software and code

Policy information about availability of computer code

Data collection	LC-MS (ThermoFisher Vanquish Flex coupled to Thermo Fisher Orbitrap Exploris 120) was used for collection of comparative untargeted metabolomic data using Thermo Scientific Xcalibur 4.4.16.2 software for data acquisition.
Data analysis	Graph Pad Prism 6, Microsoft Excel 2007, ImageJ 1.53q<br>Compound Discoverer 3.2 software was used for untargeted analysis, peak identification and integration of mass spectrometric data, MetaboAnalyst 5.0 was used for statistical analysis and visualization of metabolome data.<br>Empower 3 software was used for targeted UPLC (Waters) analysis, peak identification and integration.<br>Raw mass spectrometry data of phosphoproteomics was processed with MaxQuant version 2.0.1.0, statistical analysis and imputation of missing values was performed with Perseus software version 1.6.13.0.<br>RNA-Seq of human adipose tissues: Fastp (v0.20.0), STAR algorithm (v2.7.4a), FeatureCounts (v2.0.1), DESeq2 (v1.32.0); Correlations: R packages ggpubr (v0.4.0)

For manuscripts utilizing custom algorithms or software that are central to the research but not yet described in published literature, software must be made available to editors and reviewers. We strongly encourage code deposition in a community repository (e.g. GitHub). See the Nature Portfolio guidelines for submitting code & software for further information.

Data

Policy information about availability of data

All manuscripts must include a data availability statement. This statement should provide the following information, where applicable:

- Accession codes, unique identifiers, or web links for publicly available datasets
- A description of any restrictions on data availability
- For clinical datasets or third party data, please ensure that the statement adheres to our policy

Source data are provided with this paper as Source Data file. The datasets generated and/or analysed during the current study are available from the corresponding author on reasonable request.
Phosphoproteomic data is available at PRIDE PXD032153.
The human genome database (GRCh38.p13) is publicly available.

Field-specific reporting

Please select the one below that is the best fit for your research. If you are not sure, read the appropriate sections before making your selection.

☒ Life sciences ☐ Behavioural & social sciences ☐ Ecological, evolutionary & environmental sciences

For a reference copy of the document with all sections, see nature.com/documents/nr-reporting-summary-flat.pdf

Life sciences study design

All studies must disclose on these points even when the disclosure is negative.

Sample size	In vivo group sizes were calculated using Software Power and Sample Size Calculation (G*Power Version 3.1.9.2). No sample size calculation was performed for in vitro analysis, sample sizes were determined based on our experience from previous studies.
Data exclusions	No data were excluded.
Replication	Every experiment was repeated a minimum of three times to ensure reproducibility.
Randomization	Mice were allocated randomly into experimental groups. For diet-induced obesity studies mice were allocated into experimental groups according to their genotype. Due to the nature of the cell culture experiments, randomization of the samples was not applicable.
Blinding	Since most studies were performed by individual researchers knowing the design of the studies, blinding during data collection and analysis was not performed.

Reporting for specific materials, systems and methods

We require information from authors about some types of materials, experimental systems and methods used in many studies. Here, indicate whether each material, system or method listed is relevant to your study. If you are not sure if a list item applies to your research, read the appropriate section before selecting a response.

Materials & experimental systems

n/a	Involved in the study	
☐	☒	Antibodies
☐	☒	Eukaryotic cell lines
☒	☐	Palaeontology and archaeology
☐	☒	Animals and other organisms
☐	☒	Human research participants
☒	☐	Clinical data
☒	☐	Dual use research of concern

Methods

n/a	Involved in the study	
☒	☐	ChIP-seq
☒	☐	Flow cytometry
☒	☐	MRI-based neuroimaging

Antibodies

Antibodies used	UCP1 (Cell Signalling, Cat.No. 14670S, Lot 1), ENT1 (Antibodies online, Cat.No. ABIN387941, Lot No. SA101102BK, Clone RB28016), Calnexin (EMD Millipore Corp, Cat.No. 208880, Lot No. 3517587), P-p38 MAPK (T180/Y182) (Cell Signaling, Order No.9211S, Lot25), Phospho Creb (Ser133) (Cell Signaling, Order No.9198S, Lot14), Phospho ATF2 (Thr71) (Cell Signaling; Order No.9221S; Lot7), UCP1 (custom made), secondary antibody against rabbit (SignalStain Boost IHC; Cell Signaling; Cat. No. 8114S)
Validation	Antibodies have been tested by the manufacturers. Most of these antibodies have been published/validated by many research

Validation | groups. The UCP1-antibody (Thermo Scientific; custom made) produces a similar pattern as the UCP1-antibody (Cell Signalling, Cat.No. 14670S).

Eukaryotic cell lines

Policy information about cell lines

Cell line source(s)

Stromal vascular fraction cells were isolated from BAT and WAT of C57Bl/6 mice and differentiated to brown and white adipocytes as described in Haas et al.; 2009 and Gnad et al.; 2014; hMADS were provided by C. Dani (Nice, France). Murine microvascular endothelial cells (CI-muMECS) were purchased from InSCREENeX (Cat.No.: INS- CI-1004). Human primary brown adipocytes were isolated from BAT explant obtained from tyhroid surgeries (Ethical registration no. 076/18). Human primary white adipocytes were purchased from Lonza. HEK293T cells were purchased from ATCC (CRL-3216).

Authentication

Cell lines were not authenticated.

Mycoplasma contamination

Cell lines were not tested.

Commonly misidentified lines
(See ICLAC register)

No commonly misidentified cell lines were used.

Animals and other organisms

Policy information about studies involving animals; ARRIVE guidelines recommended for reporting animal research

Laboratory animals

C57Bl6 mice (Charles River); Adiponectin-Cre mice (Jackson Laboratory, Stock No: 010803); ENT1 floxed and global KO mice were provided by H. K. Eltzschig (Department of Anesthesiology, University of Texas Health Science Center at Houston, McGovern Medical School, Houston, Texas, USA). A2A knockout animals were purchased from The Jackson Laboratory (Strain C; 129-Adora2atm1fc/J). A2B-KO mice were provided by M. Idzko, Freiburg, Germany. DIO C57Bl6 mice were purchased from The Jackson Laboratory (Jax strain 380050).
Only male mice were analyzed.

Wild animals

No wild animals were used.

Field-collected samples

No field collected samples were used.

Ethics oversight

All animal studies were approved by the by the respective local authorities including Landesamt für Natur, Umwelt und Verbraucherschutz, Nordrhein-Westfalen, Germany and Behörde für Gesundheit und Verbraucherschutz Hamburg, Hamburg, Germany. Thermoneutrality experiments were performed at the Department of Biochemistry and Molecular Cell Biology, University Medical Center Hamburg-Eppendorf, Hamburg, Germany. Experiments with DIO C57Bl6 mice were performed at Crown Bioscience San Diego, 16550 West Bernardo Dr. #525, San Diego, CA 92127 USA. Animal welfare for this study was in compliance with the U.S. Department of Agriculture's Animal Welfare Act (9 CFR Parts 1, 2 and 3) as applicable. The animal protocol was covered by the Institutional Animal Care and Use Committee (IACUC) approved animal protocol CBSD 21-013.

Note that full information on the approval of the study protocol must also be provided in the manuscript.

Human research participants

Policy information about studies involving human research participants

Population characteristics

1. Analysis of the SLC29A1 Ile216Thr (rs45573936)variant in human subjects:
The Sorbs are of Slavonic origin, and lived in ethnic isolation among the Germanic majority during the past 1100 years. Today, the Sorbian-speaking, Catholic minority comprises 15,000 full-blooded Sorbs resident in about 10 villages in rural Upper Lusatia (Oberlausitz), Eastern Saxony. Phenotyping included standardized questionnaires for past medical history and family history, measurement of anthropometric data [weight, height, waist-to hip ratio (WHR)], and an oral glucose tolerance test (OGTT). Among the 895 available study samples (536 women and 359 men), women had a mean age of 46±16 (SD) years and a mean BMI of 26.27±5.29 kg/m2. Men had a mean age of 46±16 years and a mean BMI of 26.74±3.48 kg/m2.

2. Analysis of human visceral and subcutaneous white adipose tissues:
The human cohort comprises adipose tissues (AT) from 2,044 individuals of the Leipzig Obesity Biobank (LOBB). Omental visceral AT (VIS) samples were collected from 1,581 individuals classified as either normal weight (n = 58, mean age 60.5 ±14.8 years, mean BMI 22.5±1.9 kg/m2), overweight (n =56, mean age 65.0±12.7 years, mean BMI 27.2 ± 1.4 kg/m2), or obese (n = 1,467, mean age 47.1±11.7 years, mean BMI 48.8±8.4 kg/m2). Abdominal subcutaneous AT (SC) samples with normal weight (n = 47, mean age 64.5±13 years, mean BMI 22.9±1.7 kg/m2), overweight (n = 56, mean age 62.7±13.5 years, mean BMI 27.3±1.5 kg/m2), or obesity (n = 1,372, mean age 47.1±12 years, mean BMI 48.8 ± 8.6 kg/m2) were obtained from 1,475 individuals. Of these, paired SC and VIS data are from 1,013 patients. AT samples were collected during elective laparoscopic abdominal surgery as described {Langhardt, 2018 #2931}, immediately frozen in liquid nitrogen, and stored at -80 °C.

Recruitment

1. Analysis of the SLC29A1 Ile216Thr (rs45573936)variant in human subjects:
A convenience sample of this population was collected including unrelated subjects as well as families between 2005 and 2007. According to the exclusion criteria age below 18 years, pregnancy or lactation period, acute infections and diabetes, 895 subjects with available genotypes were included in the analyses.

2. Analysis of human visceral and subcutaneous white adipose tissues:
The human cohort comprises adipose tissues (AT) from 2,044 individuals of the Leipzig Obesity Biobank (LOBB).

For the analyses of this paper the cohorts were re-analyzed or re-genotyped, therefore, self selection bias can be excluded.

Ethics oversight

1. Analysis of the SLC29A1 Ile216Thr (rs45573936)variant in human subjects:
The study has been approved by the ethics committee of the University of Leipzig (Reg. No.: 088-2005) and is in accordance with the declaration of Helsinki. All subjects gave written informed consent before taking part in the study.

2. Analysis of human visceral and subcutaneous white adipose tissues:
The study was performed in agreement with the Declaration of Helsinki and approved by the Ethics Committee of the University of Leipzig (approval number: 159-12-21052012). All participants gave written informed consent before taking part in the study.

Note that full information on the approval of the study protocol must also be provided in the manuscript.

This checklist template is licensed under a Creative Commons Attribution 4.0 International License, which permits use, sharing, adaptation, distribution and reproduction in any medium or format, as long as you give appropriate credit to the original author(s) and the source, provide a link to the Creative Commons license, and indicate if changes were made. The images or other third party material in this article are included in the article's Creative Commons license, unless indicated otherwise in a credit line to the material. If material is not included in the article's Creative Commons license and your intended use is not permitted by statutory regulation or exceeds the permitted use, you will need to obtain permission directly from the copyright holder. To view a copy of this license, visit http://creativecommons.org/licenses/by/4.0/
