# Supplementary figures and images for: Apoptotic brown adipocytes enhance energy expenditure via extracellular inosine
Source: Nature. 2022 Jul 5;609(7926):361–8. doi: 10.1038/s41586-022-05041-0 (PMC9452294; doi:10.1038/s41586-022-05041-0)

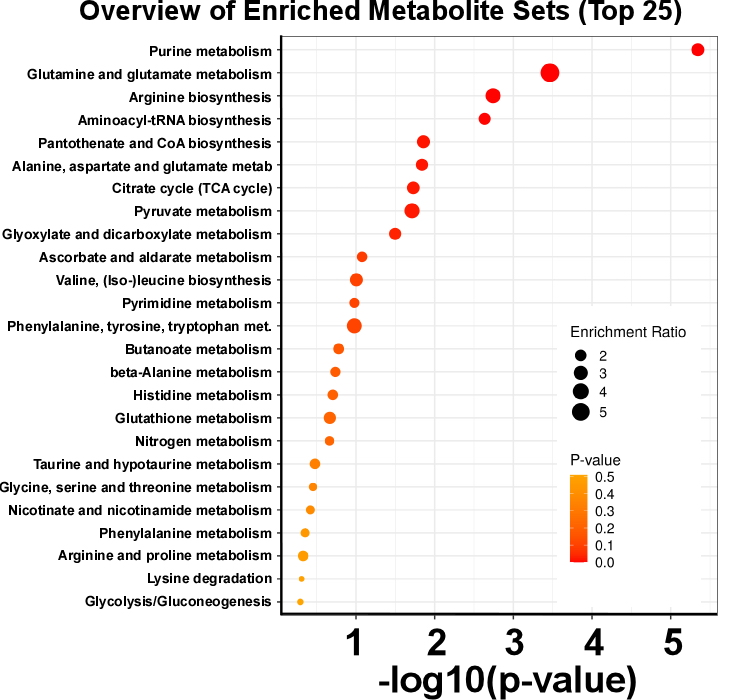

Supplement: Supplementary file 3 — Qualitative enrichment analysis of metabolic pathways. Untargeted metabolomics of murine brown adipocytes after nutlin-3 treatment: qualitative enrichment analysis of metabolic pathways of secreted metabolites based on the Kyoto Encyclopedia of Genes and Genomes metabolic pathways (n = 6). One-way ANOVA with Tukey’s post hoc test. [file 41586_2022_5041_MOESM3_ESM.jpg]
