# Supplementary material for: Apoptotic brown adipocytes enhance energy expenditure via extracellular inosine
Source: Nature. 2022 Jul 5;609(7926):361–8. doi: 10.1038/s41586-022-05041-0 (PMC9452294; doi:10.1038/s41586-022-05041-0)
Supplement: Supplementary file 4 — This file contains Supplementary Tables 1–3, which list murine primer sequences, human primer sequences and TaqMan assays. [file 41586_2022_5041_MOESM4_ESM.pdf]

Supplementary Table 1 List of murine primer sequences

Target	Forward (5´- 3´)	Reverse (5´- 3´)
Ada	GCGGTTGTTCGCTTCAAGA	GAGGAAGCTTGACTTCGCTG
Adipoq	GCCGTTCTCTTCACCTACGA	CATACACCTGGAGCCAGACTT
Cox8b	GAACCATGAAGCCAACGACT	GCGAAGTTCACAGTGGTTCC
Entpd1	ACGGACCTGGAAGAATTGGA	GTACAGGTTGGTGTGAGATGAC
Fabp4	GCGTGGAATTCGATGAAATCA	CCCGCCATCTAGGGTTATGA
Hprt	GTCCCAGCGTCGTGATTAGC	TCATGACATCTCGAGCAAGTCTTT
Nd5	AGCATTCGGAAGCATCTTTG	TTGTGAGGACTGGAATGCTG
Ndufa1	AGACGCATCTCTGGTGTCAA	GCCAGGAAAATGCTTCCTTA
Necdin	CACTTCCTCTGCTGGTCTCC	ATCGCTGTCCTGCATCTCAC
Nt5e	AAGCTATCTGGCCAACGGT	GCTGCAGAGAACTTGATCCG
Pnp	CACCTACGTGATGTTGGCAG	CCATGTGATTGGCCTTCTCC
Pparg	ACAAGACTACCCTTTACTGAAATTACCAT	TGCGAGTGGTCTTCCATCAC
Ppargc1a	GCACACACCGCAATTCTCCCTTGTA	ACGCTGTCCCATGAGGTATTGACCA
Prdm16	AGACCGAAGACGGCATCCT	CGTAGCTCGAAGTCTGGTGG
Slc29a1	AGTCACCAGCCTCAGGACAG	GGACACGTCCAGGCGGTT
Ucp1	GGCCTCTAGGACTCAGTC	TAAGCCGGCTGAGATCTTGT
Xo	GGACAACGGTAGATGAGTTGG	GAAGGCGGTCATACTTGGAG

Supplementary Table 2 List of human primer sequences

Target	Forward (5´- 3´)	Reverse (5´- 3´)
ADA	GGTCCATCCTGTGCTGCAT	TGGACAGTACGGTGAATGCC
ENTPD1	GAGGAAGGTGCCTATGGCTG	GTCCTTGCCATAGAGGCGAA
FABP4	TCAAGAGCACCATAACCTTAGATGG	GACGCATTCCACCACCAGTT
HPRT	CCTGGCGTCGTGATTAGTGA	CGAGCAAGACGTTCAGTCCT
NT5E	GAACCTGGCTGCTGTATTGC	TACACCACATGGATTCCGCC
PNP	TACGACCGGACTATGAGGCA	TGTACTCATGCCAACAGCGT
PPARG	ACAATGCTGGCCTCCTTGAT	TTGCCAAGTCGCTGTCATCT
PPARGC1A	TACTTCAGCGAGAAGCAGGC	TCACTGCACCACTTGAGTCC
PRDM16	TACTGACGGACGTGGAAGTG	GTGAGGTTCTGGTCATCGCA
TFAM	AGAACAGCTAACTCCAAGTCAGA	ACGTTATAAGCTGAACGAGGTCT
UCP1	GGAACAATCACCGCTGTGGT	ATCCTGAGAGAGGCGGAGCT
XO	CCGTCTATGCGGCTTGTCA	TGATCTCCTGTTAGGCAGTCG

Supplementary Table 3 List of TaqMan assays

TaqMan assay for Casp3	Thermo Fisher	Mm00438045_m1
TaqMan assay for Casp7	Thermo Fisher	Mm00432322_m1
TaqMan assay for Bcl2	Thermo Fisher	Mm00477631_m1
TaqMan assay for Ddit3	Thermo Fisher	Mm00492097_m1
TaqMan assay for mXbp1SV_AbD	Thermo Fisher	MXBP1SV-SV
TaqMan assay for Bax	Thermo Fisher	Mm00432050_m1
TaqMan assay for 36b4	Thermo Fisher	Mm01974474_gH
